# Psychological, economic, and ethical factors in human feedback for a chatbot-based smoking cessation intervention

**DOI:** 10.1038/s41746-025-01701-3

**Published:** 2025-05-31

**Authors:** Nele Albers, Francisco S. Melo, Mark A. Neerincx, Olya Kudina, Willem-Paul Brinkman

**Affiliations:** 1 Department of Intelligent Systems, Delft University of Technology, Delft, Netherlands; 2 INESC-ID and Instituto Superior Técnico, Universidade de Lisboa, Lisbon, Portugal; 3 Department of Values, Technology and Innovation, Delft University of Technology, Delft, Netherlands

**Keywords:** Medical ethics, Lifestyle modification, Human behaviour

## Abstract

Integrating human support with chatbot-based behavior change interventions raises three challenges: (1) attuning the support to an individual’s state (e.g., motivation) for enhanced engagement, (2) limiting the use of the concerning human resources for enhanced efficiency, and (3) optimizing outcomes on ethical aspects (e.g., fairness). Therefore, we conducted a study in which 679 smokers and vapers had a 20% chance of receiving human feedback between five chatbot sessions. We find that having received feedback increases retention and effort spent on preparatory activities. However, analyzing a reinforcement learning (RL) model fit on the data shows there are also states where not providing feedback is better. Even this “standard” benefit-maximizing RL model is value-laden. It not only prioritizes people who would benefit most, but also those who are already doing well and want feedback. We show how four other ethical principles can be incorporated to favor other smoker subgroups, yet, interdependencies exist.

## Introduction

Suppose Alice, Bob, Charlie, and 163 others are trying to quit smoking with an eHealth application. This is a sensible approach as meta-analyses on eHealth applications show that they are effective for quitting smoking^1,2^ and changing other lifestyle behaviors^3^ by providing support anywhere anytime^4^. Now, in such an eHealth application, the virtual coach Kai can support Alice, Bob, Charlie, and the other people wishing to quit. Next to Kai, the human coach Hannah can give additional feedback to increase people’s adherence to the virtual coach intervention. Since Hannah has agreed with her manager that she only has time to give 6-min feedback to around 60 clients per day, she every day needs to choose clients to help. To increase everybody’s chance of successfully quitting, it might be ideal to give feedback to all three clients alternately. However, Hannah expects, for example, that Alice would most benefit from the feedback, so she is tempted to give more feedback to Alice. On the other hand, Alice is already doing very well, whereas Bob is struggling with the intervention. So even though Bob would benefit less than Alice, Hannah feels some obligation to help him so that he also has a chance to succeed at quitting smoking. But to make it more complicated, Bob told Hannah that he does not appreciate human feedback, so she feels like she should respect his wish. This leaves Charlie: he expressed high appreciation for human feedback, but Hannah thinks that the human feedback will mainly distract him from the virtual coach intervention. So, who should Hannah give feedback to?

The question about good allocation of health professionals’ time is not a trivial one, with projections indicating that by 2060, healthcare expenses will need to rise to 18% of the Dutch GDP^5^ and one in three people will need to work in healthcare to support the aging population^6^. There is thus an evident need to make healthcare more scalable and cost-effective. One promising way are eHealth applications^7^ which provide elements of healthcare over the Internet or connected technologies such as apps and text messaging and thus reduce the need for scarce and costly human healthcare staff. Since 9.4% of the disease burden in countries such as the Netherlands results from smoking^8^, eHealth applications for quitting smoking are especially welcome. While these applications commonly integrate conversational agents that take the role of virtual coaches^9^, combining virtual with human support can be effective. Such human support can complement the strengths of virtual coaches, not only in terms of responsibility, risk, and oversight^10^, but also by providing more tailored support^11,12^, addressing things other than health in people’s lives^11^, and being more empathetic^11,12^. Human support can also lead to higher credibility^13^, which may make application features such as personalization more effective^14^. Moreover, people may be more engaged^15^ and feel more accountable^11,12,16^ and satisfied^17^ when a human coach is involved, which may help address the dropout eHealth applications often suffer from^18–20^. However, too much human support can reduce motivation, one’s sense of self-worth, autonomy, and opportunities for learning^21^. In light of these considerations, we want to examine the effects of human support in a chatbot-based intervention for quitting smoking (*RQ1*). We are specifically interested in its effects on engagement due to the central role engagement plays in intervention effectiveness^22^. Thereby, before delving into long-term effects, we will first concentrate on the short-term effects.

Whether human support is effective may depend on how motivated, confident, or tired a person is (i.e., their state). This state refers to a person’s condition or status at a specific moment in time, characterized by relative stability in its components^23^. For example, people with high intrinsic motivation who are already adhering to and engaging with the intervention might perceive human support as controlling or questioning their ability or competence^24^. At best, providing human support to people with high intrinsic motivation only makes it ineffective, but it can also undermine people’s intrinsic motivation and thus lead to lower adherence in the future^24^. So, whether human support is given in a person’s *current* state can also affect a person’s *future* state and thus the effectiveness of future human support. One approach that allows us to consider both current and future states is reinforcement learning (RL)^25^. While RL has previously been used to allocate human support in eHealth applications, the algorithms tend not to consider people’s future states (e.g., refs. ^26,27^) and current states (e.g., refs. ^28,29^). With such a consideration of current and future states, RL for adaptive behavior change support^30^ has previously been applied to various domains, such as timing running notifications^31^, suggesting step goals^32,33^, selecting messages for diabetes prevention^34^, and choosing persuasive messages for preparing for quitting smoking^35^. Here, we investigate whether RL with a consideration of current and future states can also be used to allocate human support for long-term effectiveness. Our second research question thus concerns how effective human support for quitting smoking is in the long term (*RQ2*), again with a focus on engagement.

One crucial difference when allocating human support compared to adapting other elements of eHealth applications is that human support is limited: Since one of the main motivators for creating eHealth applications is that they reduce the need for scarce and costly healthcare staff, adding large amounts of human support to eHealth applications defeats this purpose. Current eHealth applications commonly provide human support on demand (e.g., refs. ^15,36–38^). While this does not explicitly limit the amount of human support, many people do not use optional human support^21^ due to reasons such as preference for self-management^12^ or lack of perceived usefulness^12,37^ or time^37^. Therefore, since the requested amount remains relatively low in practice, limits such as maximum amounts of support per person (e.g., up to three text messages per day^37^) may not be necessary. A downside of this approach is that people who do not ask for support may still benefit from it^21^. To address this, some applications supplement the on-demand support with a certain minimum level of human support per person (e.g., refs. ^15,36,37^). This, in turn, has the disadvantage that support might be allocated to people who do not benefit or even are put off by it. Current RL algorithms for allocating human support^26–29^ do allocate human support to those who would most benefit from it by optimizing for measures such as meeting calorie goals^28^ or reducing opioid analgesic misuse risk^27^. However, they do not necessarily respect people’s autonomy by not assigning support to people who do not want it, which brings us back to the idea of providing human support on demand. Moreover, unlike applications providing a certain amount of support to each person, current RL algorithms do not ensure equal treatment by assigning the same amount of support to everybody.

Evidently, allocating limited human smoking cessation support requires moral considerations regarding who gets to benefit from human support, and thus who may increase their chance of successful smoking cessation and positive health outcomes. More generally, the question of allocating limited human support can be seen as a problem of allocating scarce medical resources, for which Persad et al.^39^ formulated four categories of ethical principles: (1) treating people equally, (2) favoring the worst-off, (3) maximizing the total benefit of the client population, and (4) promoting and rewarding social usefulness (Table 1). Each of these categories can be implemented in different ways. For example, treating people equally could mean allocating support randomly or on a *first-come, first-served* basis^39^. In addition, resource allocation differs in whether it respects people’s autonomy. According to self-determination theory, the satisfaction of autonomy together with competence and relatedness enhances motivation and well-being^40^. Moreover, autonomy is, besides justice, non-maleficence, and beneficence, one of the four main principles of biomedical ethics^41^. Goodman and Houk^42^ argue that a patient should have the ultimate say in whether to proceed with a treatment they are offered if their autonomy is to be respected. Applied to our context, this could mean that a person who does not want human support should not be given support.

**Table 1:** Allocation principles by Persad et al.^39^, with the addition of autonomy^40,41^, and corresponding examples in the context of allocating human support for preparing for quitting smoking

Allocation principle	Example for preparing to quit smoking
Treating people equally	
Lottery	- Random allocation
First-come, first-served	- Longest time since last human support
Favoring the worst-off: prioritarianism	
*Sickest first*	- Least likely to successfully prepare for quitting smoking without human support
	- Most likely to experience negative consequences of smoking in the future without human support
Youngest first	- Youngest first
Maximizing total benefits: utilitarianism	
Prognosis	- Largest increase in the chance of successfully preparing for quitting smoking because of the support
	- Largest reduction in the negative consequences of smoking in the future because of the support
Promoting and rewarding social usefulness	
Instrumental value	- Largest value to society in the future (e.g., healthcare staff, workers producing influenza vaccine, people who agree to improve their health and thus use fewer resources in the future)
Reciprocity	- Past usefulness or sacrifice (e.g., past organ donors, people who participated in vaccine research, people who made healthy lifestyle choices that reduced their need for resources in the past)
Respecting autonomy	
Autonomy	- Highest appreciation of human support

Persad et al.^39^ claim that no single principle is sufficient to include all morally relevant considerations. However, it is not obvious how the principles should be combined. Even in the intensive care unit (ICU) triage context, which has been well-studied during the COVID-19 pandemic, guidelines differ between countries^43^. For example, while maximizing benefits is in many countries a central principle in triage decisions, countries disagree on whether some priority should be given to younger people regardless of medical arguments^43^. Before deciding how to allocate human support for quitting smoking, it would help to first better understand the consequences of using different ethical allocation principles, and combinations thereof, for various subgroups. Importantly, the focus here is on first *understanding* the effects of including a wide range of different principles rather than *proposing* that certain principles should be used. Therefore, our third research question is what ethically (ir)responsible consequences may occur from using the learning algorithm and can be mitigated with algorithmic refinements (*RQ3*).

**Fig. 1:**
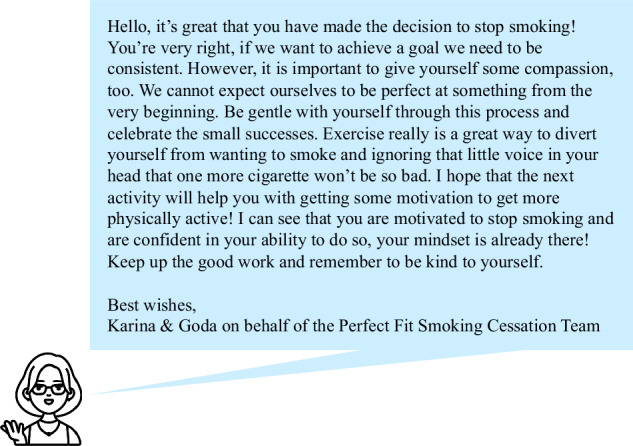
Example of human feedback sent to participants in our intervention. Participants received the human feedback messages through Prolific Academic.

The context in which we investigate our three research questions is preparing for quitting smoking or vaping with a virtual coach. Specifically, we envision a virtual coach that prepares people for quitting smoking or vaping by assigning them preparatory activities such as visualizing one’s desired future self or thinking of strategies for dealing with cravings. The goal of these activities is to prepare people for change, which is often done at the start of a behavior change intervention (e.g., refs. ^44–46^) to increase the likelihood of successful change thereafter. We focus on this first part of a behavior change intervention since feedback effects are more difficult to assess in a complete behavior change intervention with many other (adaptive) elements such as goal-setting or social support^47^. In the few days between sessions with the virtual coach, smokers may sometimes receive a feedback message from a human coach to motivate and keep them engaged (Fig. 1). To assess the effect of the feedback, the virtual coach asks smokers about the effort spent on their activities in the next session. The choice of who receives feedback is thereby guided by psychological (i.e., factors describing an individual’s state that influence the effects of human feedback), economic (i.e., cost of human feedback), and ethical (i.e., which ethical principles are used) concerns.

Since the effectiveness of eHealth applications for behavior change hinges on user engagement^22^, we here examine whether receiving human feedback increases people’s engagement with the preparatory activities proposed by the virtual coach, specifically, the effort spent on the activities and the likelihood of returning to the next session. Therefore, the effects of the intervention on actual smoking cessation lie outside the scope of our study. For our analyses, we use 2326 interaction samples from a longitudinal study in which 679 daily smokers and vapers interacted with a text-based virtual coach in up to five sessions and sometimes received human feedback between sessions. Moreover, we perform human data-based simulations with an RL model to examine the long-term effects of human feedback given in different states and under varying cost settings. Even this “standard” RL model is value-laden. It prioritizes people who are already doing well and want feedback. We conclude by showing that building an RL model for allocating limited human feedback necessitates making ethical decisions and illustrating how different ethical principles can be incorporated to favor different smoker subgroups. For this, we use the principles by Persad et al.^39^ with the addition of autonomy to capture a wide range of concrete principles used in practice. Despite the breadth of these principles, we do not exclude the possibility that other principles could be relevant in this context. To combine principles, we can use weights. For example, a total priority score could be computed as ${0.8} \times {prognosis} + {0.2} \times {youngest}\, {first}$, thereby assigning a weight of 0.8 to the benefit someone would receive from human support, a weight of 0.2 to their age-based priority, and a weight of 0 to their priorities based on any other principle. Rather than proposing optimal weights, our goal is to show the influence of different weights and, in particular, weights based on smokers’ preferences. For the latter, we use the preferences of the 449 participants of our post-questionnaire. We hope that our work helps eHealth application designers make the ethical decisions needed for allocating human support. To make our findings accessible to a broader audience, a lay summary can be found in the Supplementary Information.

## Results

We collected 2326 interaction samples from 679 people. On a scale from 0 (“nothing”) to 10 (“extremely strong”), participants reported spending an average effort of 5.74 (SD = 2.75) on their activities, ranging from 4.80 (SD = 2.72, *N* = 71) for the activity “Role model for others by quitting smoking” to 6.62 (SD = 2.42, *N* = 21) for the activity “How friends and/or family will receive one’s desired future self after quitting smoking.” The mean effort scores for all 37 activities can be found in Supplementary Table 1. In sessions 2–5, participants were asked about their likelihood of having returned to the session in case of an unpaid smoking cessation program on a scale from −5 (“definitely would have quit the program”) to 5 (“definitely would have returned to this session”). The mean of these responses was 1.57 (SD = 2.73, *N* = 679) in session 2 and 2.11 (SD = 2.68, *N* = 504) in session 5, with responses from the full range of the scale in each session. Participants seem to have read most of the messages as they clicked on the reading confirmation links for 81.72% of the 465 interaction samples with human feedback. Moreover, of the 270 people in the post-questionnaire who received at least one human feedback message, 82.59% said they noticed the human feedback messages, and 81.85% that they read the human feedback messages either sometimes (11.48%) or always (70.37%). Receiving human feedback appears not to have influenced the actual return to the next session. For example, the percentage of people answering at least one state question in session 2 is 87.26% for people who received feedback after session 1 and 86.09% for those who did not.

### RQ1: short-term effects of human feedback on engagement

We can frame posterior probabilities as “bets” we can place with varying confidence levels^48^. Looking at the *direct* effect of human feedback, we can place a casual bet that human feedback increases the effort people spend on their activities (*b* = 0.08, 95% HDI = [−0.13, 0.29], *P*(*b* > 0) = 0.76, Cohen’s *d* = 0.05), whereas it is not worth betting on a positive effect for the return likelihood (*b* = 0.03, 95% HDI = [−0.15, 0.22], *P*(*b* > 0) = 0.64, Cohen’s *d* = 0.02). The effect sizes can be evaluated as less than small according to the guidelines by Cohen^49^.

Regarding the *delayed* effect of human feedback, we find that people spend more effort on their activities (*b* = 0.39, 95% HDI = [0.17, 0.62], *P*(*b* > 0) > 0.9995, Cohen’s *d* = 0.25) and are more likely to return to the next session (*b* = 0.29, 95% HDI = [0.08, 0.50], *P*(*b* > 0) > 0.995, Cohen’s *d* = 0.18) when they have received human feedback in the past (e.g., two sessions ago). The posterior probabilities can be classified as nearing certainty that the effect of having received feedback is positive for effort and a very strong bet that it is positive for the return likelihood. The effect size is small for effort and less than small for the return likelihood^49^.

We further assessed the delayed effect of *multiple* human feedback messages. Here, we can place a casual bet that having received multiple human feedback messages increases the effort people spend on their activities (*b* = 0.14, 95% HDI = [−0.21, 0.50], *P*(*b* > 0) = 0.80, Cohen’s *d* = 0.09), whereas it is not worth betting on a positive effect for the return likelihood (*b* = 0.06, 95% HDI = [−0.24, 0.37], *P*(*b* > 0) = 0.66, Cohen’s *d* = 0.04). Both effect sizes are less than small^49^.

### RQ2: long-term effects of optimally allocated human feedback on engagement

**Fig. 2:**
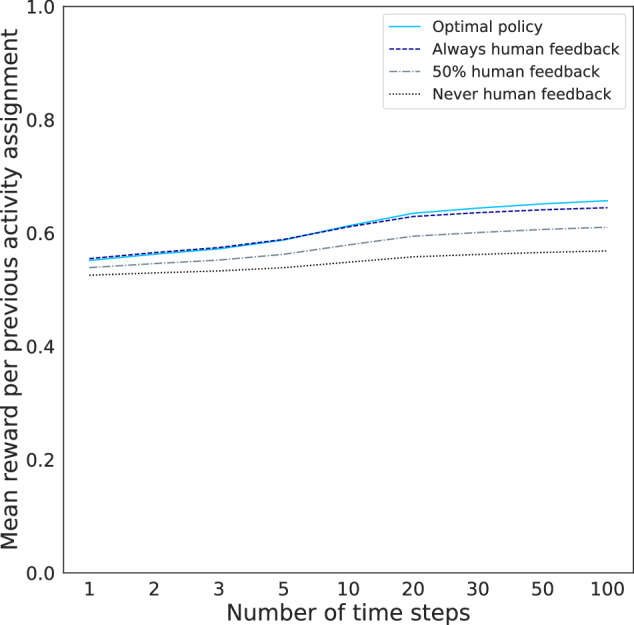
Mean reward per previous activity assignment over time for four policies. People are initially distributed across the 12 base states as in the first session of our study.

We first analyzed the long-term effects of *unlimited* human feedback. To this end, Fig. 2 depicts the mean effort-based reward per activity assignment for four policies that provide different amounts of human feedback. Comparing these policies in Fig. 2 shows that providing more human feedback *generally* leads to a higher mean reward per activity assignment. The mean rewards per activity assignment for the policies of never, half the time, and always providing feedback are 0.53, 0.54, and 0.55 after a single time step, and 0.57, 0.61, and 0.64 after 100 time steps. The latter three correspond to efforts of 6.32, 6.68, and 6.97 and thus to increases of the mean effort of 5.74 by 10.18, 16.41, and 21.52%. Always providing feedback thus ultimately leads to an effort that is by 0.65 scale points higher than never providing feedback. Looking at the optimal policy π^*,0^ in Fig. 2, however, reveals that providing more human feedback is not *always* better: the optimal policy does not always assign human feedback but ultimately leads to a higher mean reward per activity assignment than always providing human feedback. Specifically, there are two states where it is optimal not to give human feedback. In both of these states, the perceived importance of preparing to quit is high, and either the self-efficacy for preparing to quit or the human feedback appreciation is low. The mean reward per activity assignment for the optimal policy after 100 time steps is 0.66, which corresponds to an effort of 7.08 and an increase of the mean effort by 23.36%.

**Fig. 3:**
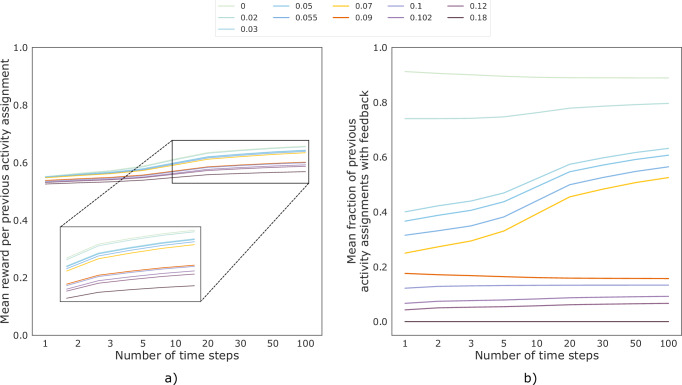
Impact of different human feedback costs on the reward and amount of allocated human feedback. Mean (**a**) reward per previous activity assignment and (**b**) fraction of previous activity assignments with human feedback for different human feedback costs. Up to costs of 0.07, large amounts of human feedback can be saved (**b**) without large drops in reward (**a**).

Regarding long-term effects of *limited* human feedback, Table 2 shows the states in which people would receive human feedback when different human feedback costs are used. States with low or medium perceived importance and high self-efficacy are those where human feedback has the most positive effect on engagement in the long run, as those states are still allocated feedback for the highest costs. Increasing the cost generally leads to only a small drop in reward (Fig. 3a) even if a lot less human feedback is given (Fig. 3b). The notable exception is increasing the cost from 0.07 to 0.09, which results in a clear drop in the mean effort spent on activities because then people who have high perceived importance, self-efficacy, and feedback appreciation (i.e., those in state 211) no longer receive feedback (Table 2).

**Table 2:** States with human feedback (*✓*) for optimal policies π^*,*c*^, computed based on different costs *c*

Policy	Low importance (0)				Medium importance (1)				High importance (2)			
	000	001	010	011	100	101	110	111	200	201	210	211
π^*,0^	*✓*	*✓*	*✓*	*✓*	*✓*	*✓*	*✓*	*✓*	*✓*			*✓*
π^*,0.02^	*✓*		*✓*	*✓*	*✓*	*✓*	*✓*	*✓*	*✓*			*✓*
π^*,0.03^			*✓*	*✓*	*✓*	*✓*	*✓*	*✓*	*✓*			*✓*
π^*,0.05^			*✓*	*✓*	*✓*	*✓*	*✓*	*✓*				*✓*
π^*,0.055^			*✓*	*✓*	*✓*		*✓*	*✓*				*✓*
π^*,0.07^			*✓*	*✓*			*✓*	*✓*				*✓*
π^*,0.09^			*✓*	*✓*			*✓*	*✓*				
π^*,0.1^			*✓*				*✓*	*✓*				
π^*,0.102^							*✓*	*✓*				
π^*,0.12^								*✓*				
π^*,0.18^												

*Note*: We refer to the 12 states with three-digit strings representing the values of the three state features: (1) perceived importance, (2) self-efficacy, and (3) human feedback appreciation.

### RQ3: effect of different ethical allocation principles on human feedback received by smoker subgroups

**Fig. 4:**
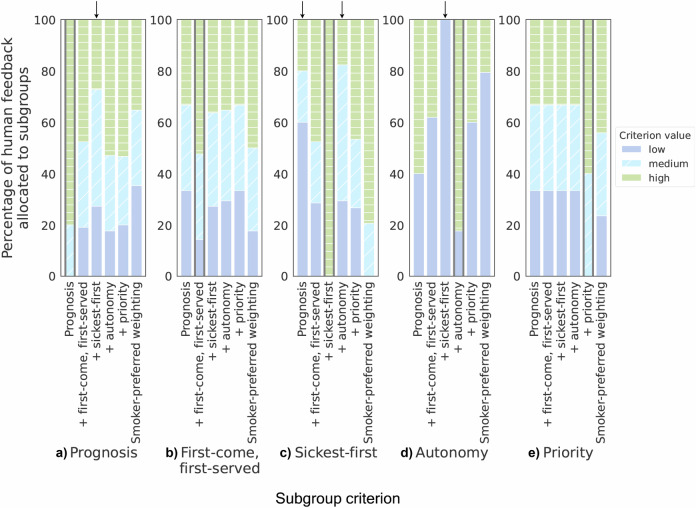
Impact of different policies on the amount of feedback allocated to different smoker subgroups. Percentage of human feedback allocated to smoker subgroups with high, medium, and low values for the criteria (**a**) *prognosis*, (**b**) *first-come, first-served*, (**c**) *sickest first*, (**d**) *autonomy*, and (**e**) *priority* when using six different policies to allocate feedback. These policies are based on either a single reward (*prognosis*), the weighted sum of two rewards (e.g., *prognosis* + *autonomy*), or the weighted sum of all five rewards using the smoker-preferred weighting shown in Table 4 (*smoker-preferred weighting*). There are only low and high values for the criterion *autonomy*. For each criterion, the bar of the policy that specifically addresses only that criterion, besides *prognosis*, is highlighted with a thick border. Arrows indicate conflicts between ethical principles.

Figure 4 depicts the percentage of human feedback allocated to smoker subgroups, distinguished based on the criteria of *prognosis*, *first-come, first-served*, *sickest first*, *autonomy*, and *priority*, when using different policies to allocate feedback. For example, when we allocate feedback using only a *prognosis*-based reward, around 80% of the feedback is given to people with a high value for the criterion *prognosis* (i.e., those most benefitting from the feedback, first bar in Fig. 4a). On the other hand, if we use a weighted combination of *prognosis* and *autonomy* to allocate feedback, only about 50% of the feedback is given to people who would benefit most from it (fourth bar in Fig. 4a). In general, Fig. 4 shows that the reward functions included in the RL model influence the way smoker subgroups are allocated feedback. While adding to the *prognosis*-based reward, a single auxiliary reward based on another ethical principle allows more feedback to be allocated according to that principle (see bars with thick borders in Fig. 4), ethical principles can be conflicting. Specifically, allocating feedback according to *sickest first* leads to much worse performance for *autonomy* and *prognosis* and vice versa (see the arrows in Fig. 4). This suggests that people who are not doing well are less likely to want and benefit from human feedback than people who are doing well. Considering smokers’ allocation preferences (Table 4), we observe that an optimal policy based on smoker-preferred principle weighting allocates less feedback to those who would most benefit from it (*prognosis*, Fig. 4a) and value it the most (*autonomy*, Fig. 4d), compared to an optimal *prognosis*-based policy. On the other hand, considering smokers’ preferences means allocating more feedback to people who have spent a lot of time since the last feedback (*first-come, first-served*, Fig. 4b), are expected to spend the lowest effort on their activities without feedback (*sickest first*, Fig. 4c), and have an individual characteristic-based priority (*priority*, Fig. 4e). This shows that smokers’ preferences differ from what is optimal when we just strive to optimize population-level health outcomes. Free-text descriptions of smokers’ allocation preferences from our post-questionnaire underline this. For example, one participant wrote, “Help those first who need the most help, but also be equal, like give feedback at least once to each person.” Supplementary Table 4 shows for each principle a quote from a participant who afterward indicated a relatively high preference for the principle.

## Discussion

The presented study examined the psychological, economic, and ethical factors that arise when combining human support in the form of human feedback messages with a chatbot-based smoking cessation intervention. While our inferential analyses provide only limited support for a positive *direct* effect of human feedback on the effort people spend on their activities and their return likelihood, they do provide strong support for a positive *delayed* effect, albeit one that is at most small (*RQ1*). That is, people who have received human feedback at some point in the past (e.g., two sessions ago) spend more effort and are more likely to return to the next chatbot session. Such small positive effects of human support have also been found in other contexts, such as mental health^50,51^. Our observed delayed effect instead of a direct effect makes sense in the tradition of primarily seeing human support as enhancing accountability or adherence^21,24^: human feedback received in the past also affects accountability at least in the short-term future. It would be interesting to see whether more direct effects are observed for different kinds of support, such as one that encourages curiosity^52^ or aims to deepen the skills or knowledge taught in an activity^21^.

Beyond the effect of having received human feedback, we only find limited support for a positive effect of having received *multiple* feedback messages. This is in line with previous work in the context of an Internet-based intervention for panic disorder^53^, a web- and mobile app-based mental health promotion intervention^54^, and a computerized psychological intervention for comorbid mental health and substance use problems^55^. Thus, more human support beyond some minimal level of human involvement might not have additional benefits. Notably, even people who never received human feedback in our study spent a somewhat higher effort (*M* = 5.83, SD = 2.43, 95% HDI = [5.49, 6.17]) than participants of our two previous chatbot-based smoking cessation studies without human involvement^56,57^ (*M* = 5.60, SD = 2.56, 95% HDI = [5.43, 5.78]) on activities common to all three studies. Therefore, future research on the effect of merely knowing that a human *could* give feedback is warranted.

Regarding long-term effects, simulations with an RL model show that while providing more human feedback generally leads to a higher effort-based reward over time, there are also two states where it is better in the long run not to provide feedback (*RQ2*). These are states where the perceived importance is high, and either self-efficacy or the appreciation of human feedback is low. This underlines that whether providing human support positively affects engagement in the long run depends on a person’s state, described by psychological factors. While our examined psychological factors primarily relate to engagement, future research could also examine factors related to other reasons why people may fail to benefit from a behavior change application and hence benefit from human support (e.g., lack of knowledge on how to use the intervention)^21^. Interesting is also that even in states where human feedback appreciation is low, providing human feedback is often beneficial in the long run (Table 2). While not receiving one’s preferred type of support does not necessarily negatively influence adherence and effectiveness^54^, it could also be that people who are strongly opposed to certain types of feedback drop out at the very start of the intervention^54^. Such self-selection might also have occurred in our study. Since we observed a median human feedback appreciation rating of 6 on a scale from –10 to 10, this seems likely.

While it is optimal to give human feedback in almost all states, about 50% of human feedback can be removed without a large drop in effort spent on activities (Fig. 3). When the human feedback cost is high, the largest long-term increase in effort can be obtained by giving feedback to people with low or medium perceived importance of preparing for quitting and high self-efficacy, which suggests an importance-enhancing effect of human feedback (Supplementary Fig. 1). While the cost of our human support was already relatively low because our human coaches were Master’s students in Psychology who spent only five to ten minutes per message, previous work suggests other types of support that could be used to potentially further reduce cost. For example, given that the qualification of human coaches providing support does not seem to play a large role in internet-based mental health interventions^50^, it has been proposed that technicians^58^ or expert patients^17^ could also provide feedback. Other alternatives include tips and personal stories from other users^15^ as well as group sessions^59^, even though the greater effort for users and loss of anonymity associated with the latter might lead to higher dropout^54^. Furthermore, besides changing the type of support, one could also reduce costs by examining why around 20% of people did not read feedback messages. It could be that some of them needed less feedback. Lastly, one could investigate how human coaches can write feedback more quickly. For example, it was more difficult for our human coaches to write feedback when people did not provide much information in their introduction texts and activity experiences.

More generally, however, sharing user data with human coaches raises privacy and transparency concerns^16^. For example, while our participants were told in the ethics board-approved informed consent form that human coaches could potentially read their anonymized introduction texts, activity experiences, and background information (e.g., baseline smoking/vaping frequency), we need to keep in mind that participants might not actually (fully) read or understand informed consent forms^60^. Our participants were reminded during the virtual coach sessions only that their activity experiences could be read by human coaches, so not all participants might have been aware of all the information that the human coaches could access. One way to address this is to let users see which data their human coach has access to and explicitly share data with them^15^. This, however, could mean that users share very little data, which could come at the expense of effective feedback, as the experiences of our human coaches and also the therapists in the study by Doherty et al.^15^ suggest. Explaining to users the benefit of sharing more information could help. Future research should also investigate the effect of feedback quality.

When it comes to allocating limited human feedback, the RL model that optimizes the effort spent on activities is essentially value-laden^61^, because it takes a stand on the ethical question of who should receive feedback when resources are limited. Specifically, in a setting where only around 35% of people receive feedback, the model gives less feedback to people who have spent the longest time since the last feedback (*first-come, first-served*), are least likely to engage with the intervention without feedback (*sickest first*), or should be prioritized based on individual characteristics such as age or future or past value to society (*youngest first, instrumental value, reciprocity*) than the 449 participants of our post-questionnaire would prefer (Table 4) (*RQ3*). Our results show that we can define additional reward functions to favor those people. Notably, however, we find that favoring one principle can come at the expense of another. For example, since our participants with low engagement often did not want feedback, giving more feedback to them means doing worse on *autonomy*. These conflicts between ethical principles raise the question of how allocation decisions can be made in practice. In a medical context, it has often been advocated that multidisciplinary teams decide how to allocate limited medical resources^43,62^. In case these teams are unsure of their preferences over different allocation principles, an explicitly multi-objective approach can be taken where teams are presented with and guided in choosing from the set of allocations that are optimal under different weights given to allocation principles^63,64^. To increase acceptance of the selected allocation, the underlying rationale should be explained^61,65^. Individual allocation outcomes could further be explained with directions to how other outcomes can be obtained^66^. Lastly, in case the model is continuously updated with new data collected during the intervention, approaches to normative monitoring of the model (e.g., refs. ^67,68^) might be useful to determine and potentially adjust the degrees to which allocation principles are followed.

In addition to the limitations related to the type and quality of feedback, as well as the possible self-selection of participants who favor receiving human feedback, our work is further limited in several ways. First, our RL-based analysis of the long-term effects of human feedback is based on human data-based simulations. Although this is a common way to assess RL models^30^, future work should compare the long-term effects of different ways of allocating human feedback in a randomized controlled trial to see how well our findings generalize. Such a trial could also integrate our preparatory activities into a full behavior change intervention to confirm whether higher engagement with preparatory activities is associated with more successful smoking cessation. Just like the engagement with these activities, their effectiveness may also depend on people’s states. We are currently investigating this in a separate study^57^. Analyzing the data on people’s experiences with their activities that we publish with this paper can further provide insights on when and for whom preparatory activities are effective. Additional field studies could also assess the predicted ethical implications of our analyses. Second, while our analysis of short-term effects for *RQ1* shows a delayed effect of providing feedback, it is not clear whether the state transitions in our RL model are sufficient to capture these delayed effects. Future works should investigate this. Third, since participants were paid for completing the conversational sessions, they might have felt at least some obligation to complete the activities even though they were informed that their payment was not contingent on their reported completion. As such, there might already have been some accountability to the intervention, which might have limited the additional effect of human feedback. Interestingly, feedback is allocated in fewer and different states when using the return likelihood instead of the effort as the basis for the reward (Supplementary Table 2), which underlines the importance of testing the effect of human feedback in an intervention without payments. Fourth, as our participants were relatively young and well-educated (Supplementary Table 5), our findings might not generalize to an older and less educated sample. Such a sample might, for example, benefit from more support, particularly that which also addresses low eHealth literacy and limited technology skills^69,70^. Future research could examine how our findings generalize to people who are underrepresented in our sample. Furthermore, there is potentially limited generalizability of our findings to other clinical settings. Even though similar analyses could be performed, the specific results (e.g., states in which human feedback is optimal, ways in which ethical principles are conflicting) would probably differ. Since human feedback has also been shown to be effective in other contexts such as mental health^50,51^, it is promising to investigate this further. Lastly, our findings depend on the specific ways in which we operationalized the ethical principles in our context. This operationalization alone can have ethical consequences (e.g., see Obermeyer et al.^71^ in the context of using historical health costs as a proxy for health needs).

In conclusion, using data from our longitudinal study in which 679 daily smokers and vapers interacted with a text-based virtual coach in up to five sessions and sometimes received human feedback between sessions, we demonstrate that people who have received human feedback spend more effort on the activities proposed by the virtual coach and are more likely to return to the next session. This suggests that it would be beneficial to have a human coach check in at least once with people who are preparing to quit smoking with a virtual coach. Moreover, concerning long-term effects, simulations with an RL model show that while providing more human feedback generally leads to a higher effort, there are also states where it is better not to provide feedback. When only very few resources for providing feedback are available, the highest effort spent on activities over time can be obtained by giving it to people with high self-efficacy and low or medium perceived importance of preparing for quitting smoking/vaping. Third, even the “standard” benefit-maximizing RL model is value-laden, prioritizing people who are already doing well and want feedback. This is noteworthy in times when the increasing pressure on the healthcare system leads to calls to focus more on the cost-effectiveness of healthcare^6^. We further show how the RL model can be extended to incorporate other ethical principles, such as favoring the worst-off or treating people equally, which we find to influence which smoker subgroups receive feedback. Since there is thus no value-free allocation of human support, moral decisions on who gets human support cannot be avoided. Given the complexity and dependencies between ethical principles, determining the consequences of different moral decisions is crucial. We hope that our work facilitates this and thus helps in making moral allocation decisions.

## Methods

We conducted an online crowdsourcing study in which participants interacted with the virtual coach Kai in up to five sessions between 1 February and 19 March 2024. The Human Research Ethics Committee of Delft University of Technology granted ethical approval for the study (Letter of Approval number: 3683). We preregistered the study in the Open Science Framework (OSF)^72^, and no changes were made compared to the preregistration.

### Study design

**Fig. 5:**
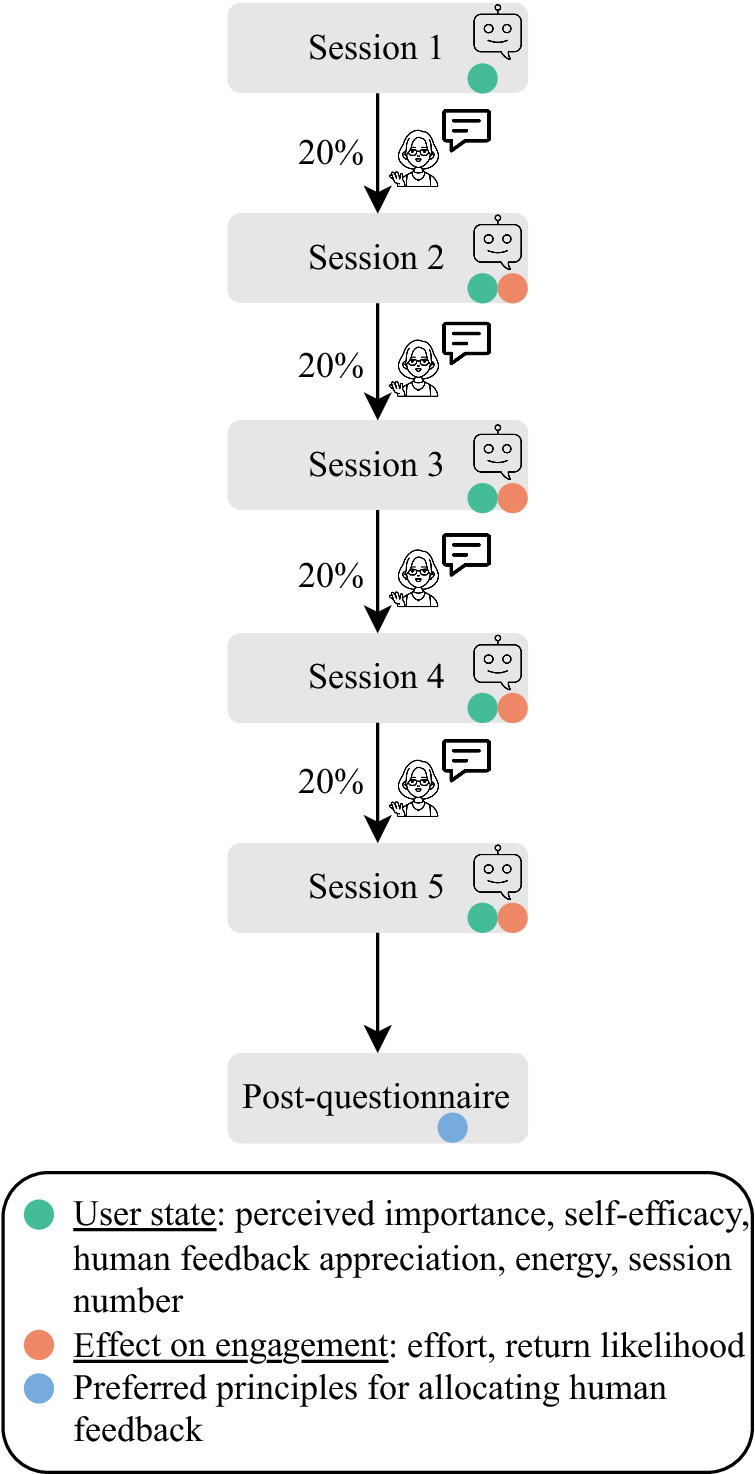
Study design, including the collected data. Between each pair of sessions, participants had a 20% chance of receiving a human feedback message.

We performed a longitudinal study with a micro-randomized design^73^, which entails assigning an intervention option at random to each participant at each pertinent decision point. The two intervention options were providing and not providing human feedback, which were chosen with probabilities of 20% and 80%, respectively. The four decision points were the days between each pair of five sessions with the virtual coach. To assess the effect of the intervention options, participants reported their effort spent on the activity assigned by the virtual coach as well as their return likelihood in case of an unpaid intervention in sessions 2–5 (Fig. 5). Based on the collected data, we performed inferential statistics to determine the effect of human feedback on the effort and return likelihood (*RQ1*). Moreover, we trained an RL model that optimizes the effort people spend on their activities over time. Using this model, we ran human data-based simulations to assess the long-term effects of human feedback under varying settings for the cost of providing feedback (*RQ2*). Such human data-based simulations are a common way to assess RL models^30^. Lastly, we compared the optimal policies of RL models that not only optimize the effort spent on activities (i.e., *prognosis*) but also account for other ethical principles (Table 4) concerning the human feedback allocated to different smoker subgroups (*RQ3*). The weights assigned to the different ethical principles are thereby also based on smokers’ preferred principles for allocating human feedback from our post-questionnaire (Table 4).

### Materials

The materials developed for this study include the virtual coach Kai, 37 preparatory activities for quitting smoking, and human feedback messages.

We implemented the text-based virtual coach Kai by closely following the implementation of the virtual coach Sam^74^, which was developed for another smoking cessation study and overall perceived positively by smokers^12,75^. There were two versions of Kai, one for smokers and one for vapers. Below, we describe the smoking version. The only difference of the vaping version is that smoking-related terms in the dialogs were replaced with their vaping counterparts (e.g., “smoker” was replaced by “vaper”). After introducing itself as wanting to prepare people for quitting smoking and becoming more physically active, with the latter possibly aiding the former^76,77^, Kai explained that one of two human coaches could sometimes send a feedback message between sessions. These human coaches were described as having a background in Psychology, including knowledge of how to help people change their behavior. In each of up to five sessions, Kai collected information on an individual’s current state by asking about their importance and self-efficacy for preparing for quitting, human feedback appreciation, and energy. Afterward, Kai proposed a new preparatory activity. In the next session, Kai asked about the effort people spent on their activity and their experience with it, as well as their likelihood of returning to the session if it was unpaid. People were told that one of the human coaches could read their experience description to write a feedback message, and that more specific descriptions would help write more specific feedback. Kai informed people when they were chosen to receive human feedback after the session. At the end of the session, participants received a reminder message with their activity on Prolific (Supplementary Fig. 2). Like Sam, Kai gave compliments for spending a lot of effort on activities, expressed empathy otherwise, and maintained an encouraging attitude. The Rasa-based implementation of Kai^78^ and a demo video^79^ are available online. The conversation structure is shown in Supplementary Fig. 3.

In each session, Kai proposed a new preparatory activity. This activity was randomly chosen from a set of 37 short activities (e.g., past successes for quitting smoking, role model for others by quitting smoking, visualizing becoming more physically active as a battle) created based on discussions with health experts, the activities of the smoking cessation applications by Michie et al.^80^ and Albers et al.^12^, the behavior change techniques by Michie et al.^81^, and smoking cessation material by organizations such as the National Cancer Institute and the Dutch Trimbos Institute. Since becoming more physically active can make it easier to quit smoking^76,77^, 17 activities addressed becoming more physically active. One example of an activity is given in Table 3 and all activities can be found in Supplementary Table 3.

**Table 3:** Title and formulation of 1 of the 37 preparatory activities for quitting smoking used in the study

**Reasons for quitting smoking**. Quitting smoking has many benefits. Think, for example, of improved physical fitness, healthier skin, and lower expenses. To help you quit smoking, it can be useful to write down why you want to quit. This can increase your aspiration to quit smoking, which may aid in quitting successfully. So, before the next session, I advise you to identify and write down reasons why you want to stop smoking. After writing them down, think about which reasons are most important to you and order them accordingly.	

**Fig. 6:**
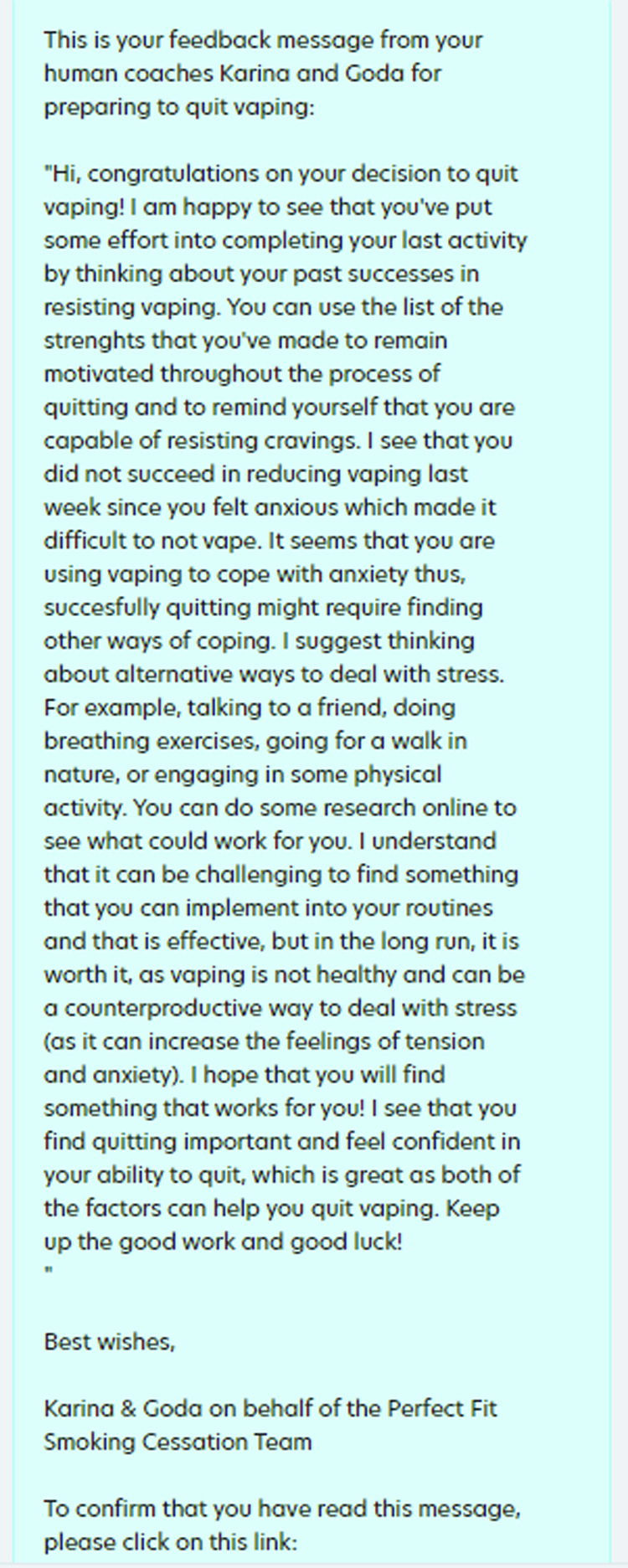
Screenshot of a human feedback message sent to a participant on the crowdsourcing platform Prolific Academic. The message ended with a disclaimer that it was not medical advice.

Between sessions, participants sometimes received a human feedback message. These messages were written by one of two human coaches, who were Master’s students in Psychology. Following the model by op den Akker et al.^82^, the human coaches were instructed to write messages that contained the following components: feedback, argument, and suggestion or reinforcement. They also received the general guidelines to refer to things in people’s lives to build rapport, show understanding if people have low confidence, and reinforce people when they are motivated. When writing the feedback, the human coaches had access to anonymized data on people’s baseline smoking and physical activity behavior (i.e., smoking/vaping frequency, weekly exercise amount, existence of previous quit attempts of at least 24 hours, and the number of such quit attempts in the last year), introduction texts from the first session with the virtual coach, previous preparatory activity (i.e., activity formulation, effort spent on the activity and experience with it, return likelihood), current state (i.e., self-efficacy, perceived importance of preparing for quitting, human feedback appreciation), and new activity formulation. All feedback messages ended with a disclaimer that they were not medical advice. A screenshot of how we sent human feedback messages to participants is provided in Fig. 6. All 523 written messages are available online^83^.

### Measures

We collected four primary groups of measures, namely, the effort spent on activities, the likelihood of returning to a session, state features, and participants’ preferred principles for allocating human feedback.

The virtual coach asked participants about the *effort* they put into their previously assigned activity on a scale from 0 (“Nothing”) to 10 (“Extremely strong”), adapted from Hutchinson and Tenenbaum^84^ as also done by Albers et al.^35^.

To determine participants’ *return likelihood*, the virtual coach asked participants the question “Currently you are taking part in a paid experiment. Imagine this was an unpaid [smoking/vaping] cessation program. How likely would you then have quit the program or returned to this session?”, rated on a scale from −5 (“definitely would have quit the program”) to 5 (“definitely would have returned to this session”). 0 was labeled as “neutral.”

Moreover, we measured five variables (i.e., features) that describe a person’s *state* in each session: (1) the perceived importance based on the question “How important is it to you to prepare for quitting [smoking/vaping] now?”, adapted from Rajani et al.^85^ and rated on a scale from 0 (“not at all important”) to 10 (“desperately important”), (2) self-efficacy based on the question “How confident are you that you can prepare for quitting [smoking/vaping] now?”, adapted from the Exercise Self-Efficacy Scale is by McAuley^86^ and rated on a scale from 0 (“not at all confident”) to 10 (“highly confident”), (3) human feedback appreciation based on the question “How would you view receiving a feedback message from a human coach after this session?”, rated on a scale from -10 (“very negatively”) to 10 (“very positively”), with 0 labeled as “neutral,” (4) energy based on the question “How much energy do you have?”, rated on a scale from 0 (“none”) to 10 (“extremely much”), and (5) the session number.

We further determined participants’ *preferred principles for allocating human feedback* by asking them to distribute 100 points across 11 allocation principles after the question, “Based on which principles/rules should the virtual coach decide when a human coach should give feedback to people who are preparing to quit [smoking/vaping]? Assign 100 credits to the principles below, where more credits mean that you are more in favor of a principle.” Nine principles were derived from those presented by Persad et al.^39^, adapted to the smoking cessation context (Supplementary Table 4). We supplemented these principles with one further formulation of treating people equally (i.e., *least amount of human feedback so far*) and with the principle of respecting people’s *autonomy* by prioritizing people who most appreciate receiving human feedback.

### Participants

Participants were recruited from the crowdsourcing platform Prolific Academic. Eligible were people who smoked tobacco products or vaped daily, were fluent in English, and had not participated in the conversational sessions of our two previous studies with similar preparatory activities^56,57^. Participants further had to give digital informed consent, confirm smoking/vaping daily, and indicate being contemplating or preparing to quit smoking/vaping^87^ and not being part of another intervention to quit smoking/vaping to pass the prescreening questionnaire. The study was framed as preparation for quitting smoking/vaping for people recruited as daily smokers/vapers. Out of 852 people who started the first conversational session, 500 completed all five sessions, and 449 provided their preferences for allocating human feedback based on different principles in the post-questionnaire. To increase the chance that participants would read the human feedback messages, they were told they might be asked to confirm having read a received message to be invited to the next session. Participants who failed more than one attention check in the prescreening questionnaire were not invited to the first session. Moreover, participants had to respond to the invitations to the sessions and the post-questionnaire within two days. The participant flow is shown in the Supplementary Information. Participants who completed a study component were paid based on the minimum payment rules on Prolific, which require a payment rate of six pounds sterling per hour. Participants were informed that their payment was independent of how they reported on their preparatory activities to account for self-interest and loss aversion biases^88^. Participants who failed more than one attention check in the prescreening or post-questionnaire were not compensated for that respective questionnaire. Participants were from countries of the Organization for Economic Co-operation and Development (OECD), excluding Turkey, Lithuania, Colombia, and Costa Rica, but including South Africa^89^. Of the 679 participants with at least one interaction sample, 330 (48.60%) identified as female, 335 (49.34%) as male, and 14 (2.06%) provided another gender identity. The age ranged from 19 to 71 (*M* = 36.30, SD = 11.21). Further participant characteristics (e.g., education level, smoking/vaping frequency) can be found in Supplementary Table 5.

### Procedure

Participants meeting the qualification criteria could access the prescreening questionnaire on Prolific, and those who passed the prescreening were invited to the first session with Kai about 1 day later. Invitations to a subsequent session were sent about 3 days after having completed the previous one. Between sessions, participants each time had a 20% chance of receiving a human feedback message. About three days after completing the last session, participants were invited to a post-questionnaire in which they were asked about their preferred principles for allocating human feedback, first by means of an open question and then by distributing points across given principles.

### Data preparation

We collected all interaction samples of pairs of sessions in which people answered at least the effort, return likelihood, and the first state feature question (i.e., perceived importance) in the next session. Missing values in interaction samples (*N* = 5) for the remaining state features were imputed with the corresponding feature’s sample population median. Our data and analysis code are publicly available^90^.

### Data analysis for RQ1: short-term effects of human feedback on engagement

First, we wanted to assess whether human feedback positively affects engagement in the short term. For this, we performed Bayesian inferential analyses.

To determine the *direct* effect of human feedback on the effort people spend on their activities and their return likelihood, we compared samples where people received human feedback to samples where they did not. For each of the two dependent variables (i.e., effort and return likelihood), we fit a model containing a general mean, a random intercept for each participant, and a binary fixed effect for human feedback received after the previous session. We fit both models with diffuse priors based on the ones used by McElreath^91^ and assessed them by interpreting the posterior probability that the fixed effect for human feedback is greater than zero based on the guidelines by Chechile^48^. We further report 95% highest density intervals (HDIs).

Besides the direct effect of human feedback on the effort and return likelihood, there might also be a *delayed* effect. For example, if human feedback increases a person’s self-efficacy, then the person may spend a lot of effort on future activities even when not receiving additional human feedback. To determine whether having received human feedback leads to a higher effort and return likelihood, we fit two further statistical models. For both dependent variables (i.e., effort and return likelihood), we fit a model containing a general mean, a random intercept for each participant, and a fixed effect for whether participants had received human feedback until then. We again fit both models with diffuse priors and used posterior probabilities and 95% HDIs to assess whether the effect of having received human feedback is positive.

The delayed effect of human feedback might be stronger for people who have received *multiple* feedback messages. To determine whether having received more human feedback leads to a higher effort and return likelihood, we created two further statistical models by extending the previous two models with a fixed effect for the number of times participants had received human feedback until then. Again, we fit both models with diffuse priors and used posterior probabilities and 95% HDIs to assess whether the effect of multiple human feedback messages is positive.

### Data analysis for RQ2: long-term effects of optimally allocated human feedback on engagement—RL model

While our inferential analysis of delayed human feedback effects already looked a few steps into the future, it was based on randomly allocated human feedback. However, in some situations, giving human feedback might also be detrimental in the long run. So now, we want to use simulations to assess the long-term effects of optimally allocated human feedback based on a person’s state. With optimally allocated human feedback, we mean feedback that is only given in situations (a) where it is ultimately more beneficial than not giving feedback, and (b) where this benefit outweighs the economic cost of giving human feedback.

**Fig. 7:**
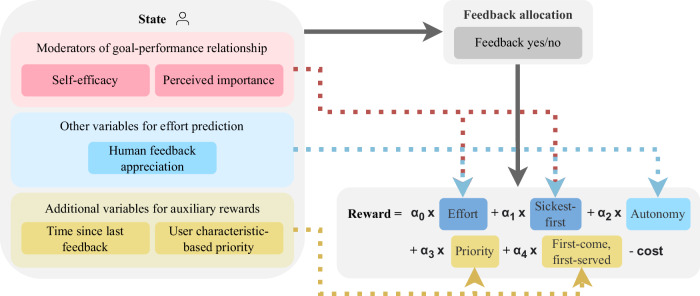
Visualization of the final RL model. The arrows indicate which state features are used to predict the different reward functions. The five reward functions can be combined by setting different weights *α*.

To study the long-term effects of optimally allocated human feedback, we designed and trained an RL model for deciding when to allocate human feedback. Starting with a base model that maximizes the effort people spend on their activities over time, we add the consideration of human feedback costs, and later for *RQ3* of other ethical principles for allocating feedback. Figure 7 visualizes our final model.

We can define our approach as a Markov decision process (MDP) 〈*S*, *A*, *R*, *T*, *γ*〉. The action space *A* consisted of two actions (i.e., giving human feedback no/yes), the reward function *R*: *S* × *A* → [0, 1] was determined by the self-reported effort spent on activities, *T*: *S* × *A* × *S* → [0, 1] was the transition function, and the discount factor *γ* was set to 0.85 to favor rewards obtained earlier over rewards obtained later as also done in previous work (e.g., refs. ^32,35^). The finite state space *S* described the state a person was in and was captured by their perceived importance of and self-efficacy for preparing for quitting smoking/vaping, as well as their appreciation of receiving human feedback. The goal of an MDP is to learn an optimal policy π^*^: *S* → Π(*A*) that describes an action to take in each state that maximizes the expected cumulative discounted reward ${\mathbb{E}}\left[\mathop{\sum }\nolimits_{t = 0}^{\infty }{\gamma }^{t}{r}_{t}\right]$. The optimal Q-value function ${Q}^{*}:S\times A\to {\mathbb{R}}$ describes the expected cumulative discounted reward for executing action *a* in state *s* and π^*^ in all subsequent states. In the following, we describe each component in more detail.

We considered six features to describe the *state space*: (1) the perceived importance, (2) self-efficacy, (3) the difficulty of the assigned activity based on the activity difficulty ratings by Albers et al.^92^, (4) energy, (5) human feedback appreciation, and (6) the session number. The first three features were considered since goal-setting theory posits that goal commitment, facilitated by importance and self-efficacy, and task difficulty are moderators of the effects that goals have on performance^93^. More precisely, low commitment and high task difficulty might make it harder for people to reach their goals, which may make human feedback more beneficial. We further included energy since it was shown to be an important predictor of the effort people spend on preparatory activities for quitting smoking in a previous study^57^. Moreover, since the novelty of the intervention may influence people’s motivation to do the activities^12^, we also captured the session number.

To reduce the size of the state space and thus create a more robust model, we selected three abstracted base state features based on our collected data. Specifically, using the G-algorithm^94^ and its adaptation by Albers et al.^35^ as inspiration, we iteratively selected the feature for which the *Q*-values for the abstracted feature values were most different. We thereby specified the first selected feature to have three and the second and third features two abstracted values. Abstract features were computed based on percentiles. For example, to create an abstract feature with two values, we set all values less than or equal to the median to 0 and those greater than the median to 1. Besides reducing the required data, selecting a subset of the state features also has the advantage that the virtual coach would in the future need to ask people fewer questions per session, which is in line with keeping smoker demands to a minimum^80^. The three selected features were (1) perceived importance with three values, (2) self-efficacy with two values, and (3) human feedback appreciation with two values. The base state space thus had size 3 × 2 × 2 = 12. We refer to the resulting base states with three-digit strings such as 201 (here perceived importance is high, self-efficacy is low, and human feedback appreciation is high). Supplementary Fig. 5 and Supplementary Fig. 6 show the mean effort and number of samples per combination of values for the three selected features.

The *action* *space* was defined by two actions: giving (*a* = 1) and not giving (*a* = 0) human feedback.

Just as in the algorithm by Albers et al.^35^, the base *reward* signal was based on asking people how much effort they spent on their previous activity on a scale from 0 to 10. Based on the sample population mean effort $\overline{e}$, the reward *r* ∈ [0, 1] for an effort response *e* was computed as follows:

1 $$r=\left\{\begin{array}{ll}\frac{e}{2\overline{e}}\qquad\qquad\qquad{if}\,{e} \,<\, \overline{e}\\ 1-\frac{10-e}{2(10-\overline{e})}\quad\quad\;{if}\,{e} \,>\, \overline{e}\\ 0.5\qquad\qquad\qquad{otherwise}.\end{array}\right.$$

The idea behind this reward signal was that an effort response equal to the mean effort was awarded a reward of 0.5, and that rewards for efforts greater and lower than the mean were each equally spaced.

The *reward and transition functions* were estimated from our data.

Due to budget constraints, the base reward may cause human feedback to be allocated to more people than can be economically afforded. To be able to reduce the amount of allocated human feedback, we introduce the *human feedback cost* *c* to be included in the reward computation depending on the action *a*:

2 $${r}_{c}=\left\{\begin{array}{ll}{r}\qquad\qquad\qquad{if}\,a=0\\ {r-c}\qquad\qquad\;{if}\,{a=1} \quad \end{array}\right.$$

We computed 0.001-optimal *policies* and corresponding Q^*^ with Gauss-Seidel value iteration from the Python MDP Toolbox. We use π^*,*c*^ to refer to an optimal policy for a certain cost *c*.

### Data analysis for RQ2: long-term effects of optimally allocated human feedback on engagement—analysis steps

First, we assume we have no economic budget constraints and can allocate as much human feedback as we wish (i.e., *c* = 0). To assess the effects of such *unlimited* human feedback over time, we ran simulations based on our collected data to compare four different policies concerning the mean reward per activity assignment over time: (1) the optimal policy π^*,0^, (2) the policy of always assigning human feedback, (3) a theoretical average policy where each of the two actions is taken $\frac{1}{2}$ times for each person at each time step, and (4) the policy of never assigning human feedback. To obtain a realistic population, the simulated people were initially distributed across the state features following the distribution we observed in the first session of our study (Supplementary Fig. 7).

In practice, budget constraints might *limit* the amount of available feedback and thus make it impossible to always allocate human feedback according to π^*,0^. To reduce the amount of allocated human feedback, we added different human feedback costs to the base reward, and assessed the resulting mean reward and amount of allocated human feedback over time. The considered costs were chosen such that the resulting optimal policies π^*,*c*^ all differ in the number of states that are allocated feedback. We again used as the starting population the distribution of people across the 12 states we observed in our study’s first session.

### Data analysis for RQ3: effect of different ethical allocation principles on human feedback received by smoker subgroups

Given that we can only provide limited human feedback, we cannot allocate human feedback to everybody. The RL models we have trained for *RQ2* all allocate human feedback to those who will see the largest increase in effort spent on preparatory activities over time because of the feedback. This can be seen as maximizing total benefits according to the allocation principle that Persad et al.^39^ call *prognosis*. However, we can also use other ethical principles in our RL model. Here, we now want to assess the effects of incorporating different ethical allocation principles on the subgroups of smokers who receive feedback.

To get a realistic assessment of the effect of incorporating different ethical allocation principles, let us first define a potential live smoking cessation application. Suppose we have an application in which people have up to nine sessions with a virtual coach, after each of which they can get feedback from a human coach. As people sometimes drop out of eHealth applications before completing them^18,95^, we assume, based on the average percentage of negative return likelihood ratings per session of our longitudinal research study, a 15% chance that people drop out of our application after each session. The spots of people who have either completed all nine sessions or have dropped out are given to new people. These new people are distributed across the 12 base states, as in the first session of our study. Taking about six minutes to write a feedback message, the human coach can give feedback to around 58 people every day. Assuming 166 spots in the application, this amounts to 35%. Therefore, the human feedback costs in our analyses were set such that, on average, about 35% of people receive feedback every day.

To also reward allocating human feedback according to ethical principles other than *prognosis*, we extended the RL model. Specifically, we created the four auxiliary (i.e., additional) rewards shown in Table 4. We use *first-come, first-served* to illustrate the effect of treating people equally. Note that the ethical principles of *youngest first*, *instrumental value*, and *reciprocity* can all be represented by setting an individual characteristic-based priority level. To compute these auxiliary rewards, we extended the state space by two features, each with three values: (1) a random individual characteristic-based priority level that remains fixed for each person and (2) time since the last human feedback. Both of these state features only influence the auxiliary reward and not the base reward (i.e., *prognosis*). Each auxiliary reward *r*_*aux*_ ∈ [0, 1] is then computed as ${r}_{aux}=\frac{aux-au{x}_{min}}{au{x}_{max}-au{x}_{min}}$, where *aux* is a person’s value for the measure underlying the auxiliary reward (e.g., the time since the last human feedback) and aux_min_ and aux_max_ are the lowest and highest possible values for the measure.

**Table 4:** Allocation principles by Persad et al.^39^, with the addition of *autonomy*, and corresponding rewards and mean weights assigned by participants of our post-questionnaire

Allocation principles	Reward	Weight
Prognosis	*Base reward*: Prioritize people who will see the largest increase in effort because of the feedback	30.82%
Treating people equally (lottery, *first-come, first-served*, least amount of human feedback so far^1^)	*First-come, first-served*: Prioritize people who have spent the longest time since the last feedback	22.18%
Sickest first	*Sickest first*: Prioritize people who would spend the lowest effort without feedback	25.34%
Youngest first, instrumental value, reciprocity	*Priority*: Prioritize people with a higher individual characteristic-based priority	13.04%
Autonomy	*Autonomy*: Prioritize people who appreciate feedback the most	8.62%

*Note:* We use *first-come, first-served* to illustrate the effect of treating people equally.

^1^ We supplemented the principles for treating people equally by Persad et al.^39^ with the principle of prioritizing people with the least amount of human feedback so far. The reason is that while Persad et al.^39^ focus on medical resources that can be allocated to each person only once, human feedback can in our context also be allocated more than once.

Using the rewards from Table 4 and the weights given to them by smokers, we compared six policies based on which states they allocate feedback: (1) the optimal policy based on the base reward, (2–5) the four optimal policies for using the base reward together with either *first-come, first-served*, *sickest first*, *autonomy*, or *priority* with the two rewards weighted based on the smoker-preferred weights, and (6) the optimal policy based on all five rewards weighted according to weights derived from smokers’ preferred principles for allocating human feedback (Table 4). Due to the relatively large drop in reward between human feedback costs of 0.07 and 0.09 observed for our analysis of the long-term effects of limited feedback (Fig. 3a), we set the human feedback cost to 0.07 for the base reward-based optimal policy, which means that after each session around 35% of people get feedback (Supplementary Fig. 8b). Since incorporating auxiliary rewards can change the amount of allocated feedback, we tuned the costs for the other policies such that these policies also allocate feedback to around 35% of people, thus allowing for a fair comparison between policies.

## Supplementary information

Supplementary Information

## Data Availability

Our data are available at 10.4121/c11b991b-0eda-4565-b7d0-6ca7fcd1cf7e.

## Code availability

Our analysis code is available at 10.4121/c11b991b-0eda-4565-b7d0-6ca7fcd1cf7e.
