# Supplementary material for: Psychological, economic, and ethical factors in human feedback for a chatbot-based smoking cessation intervention
Source: NPJ Digit Med. 2025 May 31;8:326. doi: 10.1038/s41746-025-01701-3 (PMC12126561; doi:10.1038/s41746-025-01701-3)
Supplement: Supplementary file 1 — Supplementary Information [file 41746_2025_1701_MOESM1_ESM.pdf]

Supplementary Information for "Psychological, economic, and ethical factors in human feedback for a chatbot-based smoking cessation intervention"

Nele Albers[1*], Francisco S. Melo[2], Mark A. Neerincx[1], Olya Kudina[3], Willem-Paul Brinkman[1]

[1*]Department of Intelligent Systems, Delft University of Technology, Delft, Netherlands.
[2]INESC-ID, Instituto Superior Técnico, Universidade de Lisboa, Lisbon, Portugal.
[3]Department of Values, Technology and Innovation, Delft University of Technology, Delft, Netherlands.

*Corresponding author(s). E-mail(s): n.albers@tudelft.nl;
Contributing authors: fmelo@inesc-id.pt; m.a.neerincx@tudelft.nl; o.kudina@tudelft.nl; w.p.brinkman@tudelft.nl;

This is the Supplementary Information of our paper "Psychological, economic, and ethical factors in human feedback for a chatbot-based smoking cessation intervention." Following the same structure as in the paper, we provide (more) information on:

- The mean effort and number of samples per preparatory activity (Supplementary Table 1),
- The transition probabilities without (Supplementary Figure 1a) and with (Supplementary Figure 1b) human feedback,
- The states with human feedback for different human feedback costs when using the return likelihood as the basis for the reward (Supplementary Table 2),
- Activity reminder messages sent to participants on Prolific Academic (Supplementary Figure 2),
- Structure of the five conversational sessions with the virtual coach Kai (Supplementary Figure 3),

- Preparatory activities for quitting smoking (Supplementary Table 3),
- Allocation principles in our post-questionnaire and corresponding principles by Persad et al. [1] together with the mean weights assigned to the principles by participants as well as illustrative participant quotes (Supplementary Table 4),
- Participant characteristics such as the age and gender (Supplementary Table 5),
- Participant flow (Supplementary Figure 4),
- Mean effort per action and combination of values for the three selected state features (Supplementary Figure 5),
- Number of samples per action and combination of values for the three selected state features (Supplementary Figure 6),
- Initial distribution of people across the 12 base states observed in the first session of our longitudinal study (Supplementary Figure 7), and
- The mean reward (Supplementary Figure 8a) and percentage of people receiving feedback (Supplementary Figure 8b) per time step for different human feedback costs in our potential live application.

Moreover, we provide a summary for a lay audience at the end of the Supplementary Information.

Results

Supplementary Table 1: Mean effort and number of samples for each of the 37 preparatory activities based on the 2,326 collected interaction samples.

	Preparatory activity	Effort mean (SD)	Number
1	Creating motivational slogans/quotes for quitting smoking	6.04 (2.91)	71
2	Creating motivational slogans/quotes for becoming more physically active	5.45 (3.34)	78
3	Testimonial on becoming more physically active	5.41 (3.30)	76
4	Desired future self after quitting smoking - Writing	6.06 (2.76)	70
5	Desired future self after becoming more physically active - Writing	5.25 (2.74)	72
6	Reasons for quitting smoking	6.00 (2.76)	72
7	Reasons for becoming more physically active	5.48 (2.83)	60
8	Personal rule for not smoking	5.27 (3.28)	70
9	Personal rule for becoming more physically active	5.70 (2.56)	74
10	How friends and/or family will receive one's desired future self after quitting smoking*	6.62 (2.42)	21
11	How friends and/or family will receive one's desired future self after becoming more physically active*	5.94 (2.90)	17
12	Focusing on past successes for quitting smoking	5.51 (2.73)	65
13	Focusing on past successes for becoming more physically active	5.40 (2.36)	73
14	Role model for others by quitting smoking	4.80 (2.72)	71
15	Role model for others by becoming more physically active	6.00 (2.68)	72
16	Tracking smoking behavior	6.05 (2.52)	80
17	Tracking physical activity behavior	5.12 (2.91)	74
18	Feared future self when not quitting smoking - Writing	6.21 (2.58)	70
19	Feared future self when not becoming more physically active - Writing	5.89 (2.61)	75
20	Feared future self when not quitting smoking - Picture	5.55 (2.68)	75
21	Feared future self when not becoming more physically active - Picture	5.90 (2.67)	73
22	Visualizing smoking as a battle	5.20 (2.45)	80
23	Visualizing becoming more physically active as a battle	5.73 (2.38)	60
24	Desired future self after quitting smoking - Picture	5.86 (2.54)	77
25	Desired future self after becoming more physically active - Picture	6.34 (2.45)	71
26	Education on sleep	5.58 (3.07)	79
27	Routines that cause cravings*	6.11 (3.10)	18
28	Thinking of high-risk situations and how to cope with them*	5.58 (2.07)	12
29	Alternative behaviors for cravings	6.60 (2.29)	57
30	Progressive muscle relaxation	5.32 (3.17)	75
31	Breathing exercise	6.05 (2.39)	64
32	Exchanging a passive activity for an active one	6.13 (2.45)	82
33	Thinking of solutions to barriers to becoming physically active	5.71 (2.70)	83
34	Education on recommended physical activity*	5.67 (2.90)	12
35	Plan for becoming more physically active*	5.77 (2.89)	13
36	Positive diary	5.86 (3.04)	71
37	Focusing on past success in general	6.25 (2.68)	63
	**Total**	**5.74 (2.75)**	**2326**

* Activity had another activity as prerequisite.
Abbreviations: SD, Standard deviation.

Discussion

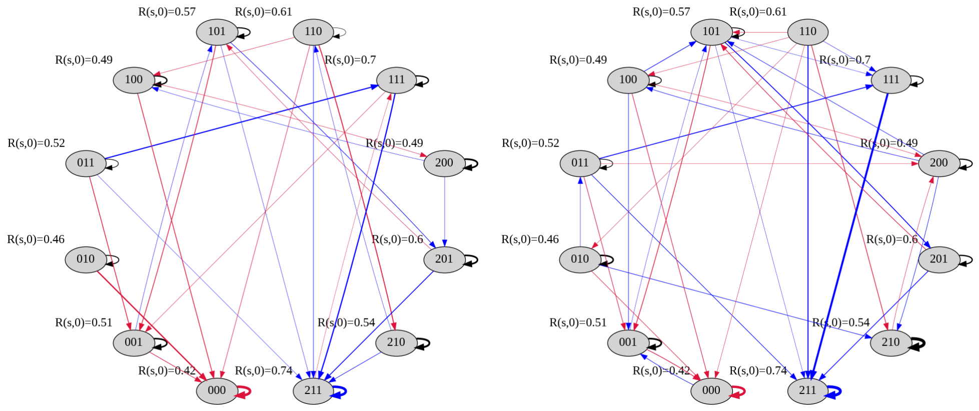

(a) Without human feedback (b) With human feedback

Supplementary Figure 1: Transition probabilities between the 12 states (a) without and (b) with human feedback. Only transitions with a probability of at least $\frac{1}{12}$ are shown. We distinguish transitions to a state with a higher or highest $R(s,0)$ (blue), lower or lowest $R(s,0)$ (red), and the same $R(s,0)$ (black), where $R(s,0)$ denotes the immediate reward when not giving feedback in state s. A thicker line denotes a higher probability.

Supplementary Table 2: States with human feedback (✓) for optimal policies $\pi^{*,c}$, computed based on different costs c, when using the return likelihood as the basis for the reward and the features selected for the effort-based reward. We refer to the 12 states with three-digit strings representing the values of the three state features: 1) perceived importance, 2) self-efficacy, and 3) human feedback appreciation.

**Policy**	**Low importance**				**Medium importance**				**High importance**			
	**000**	**001**	**010**	**011**	**100**	**101**	**110**	**111**	**200**	**201**	**210**	**211**
$\pi^{*,0}$	✓			✓	✓	✓	✓	✓	✓		✓	✓
$\pi^{*,0.01}$	✓			✓	✓	✓	✓	✓			✓	✓
$\pi^{*,0.02}$	✓			✓	✓	✓	✓	✓			✓	
$\pi^{*,0.05}$				✓	✓	✓	✓	✓			✓	
$\pi^{*,0.06}$				✓	✓		✓	✓			✓	
$\pi^{*,0.09}$				✓	✓		✓	✓				
$\pi^{*,0.137}$				✓	✓			✓				
$\pi^{*,0.14}$					✓			✓				
$\pi^{*,0.18}$								✓				
$\pi^{*,0.22}$												

Methods

Materials

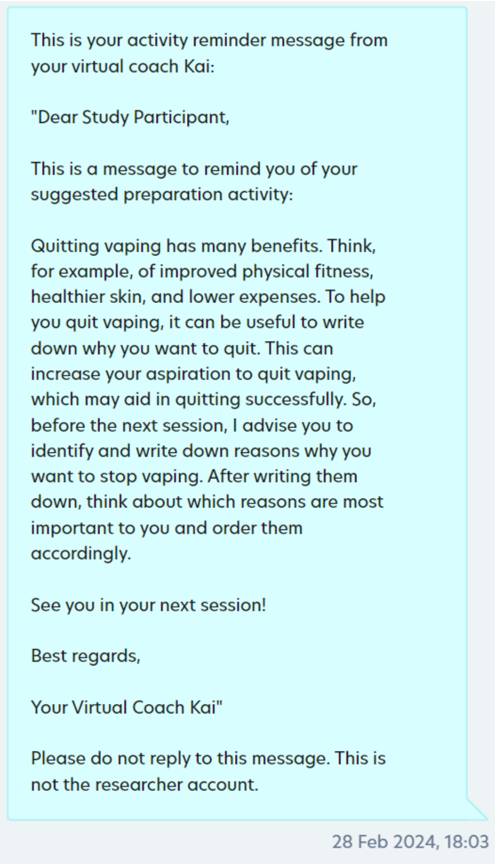

Supplementary Figure 2: Example of an activity reminder message. Screenshot of how the activity reminder messages were sent to participants on the crowdsourcing platform Prolific Academic.

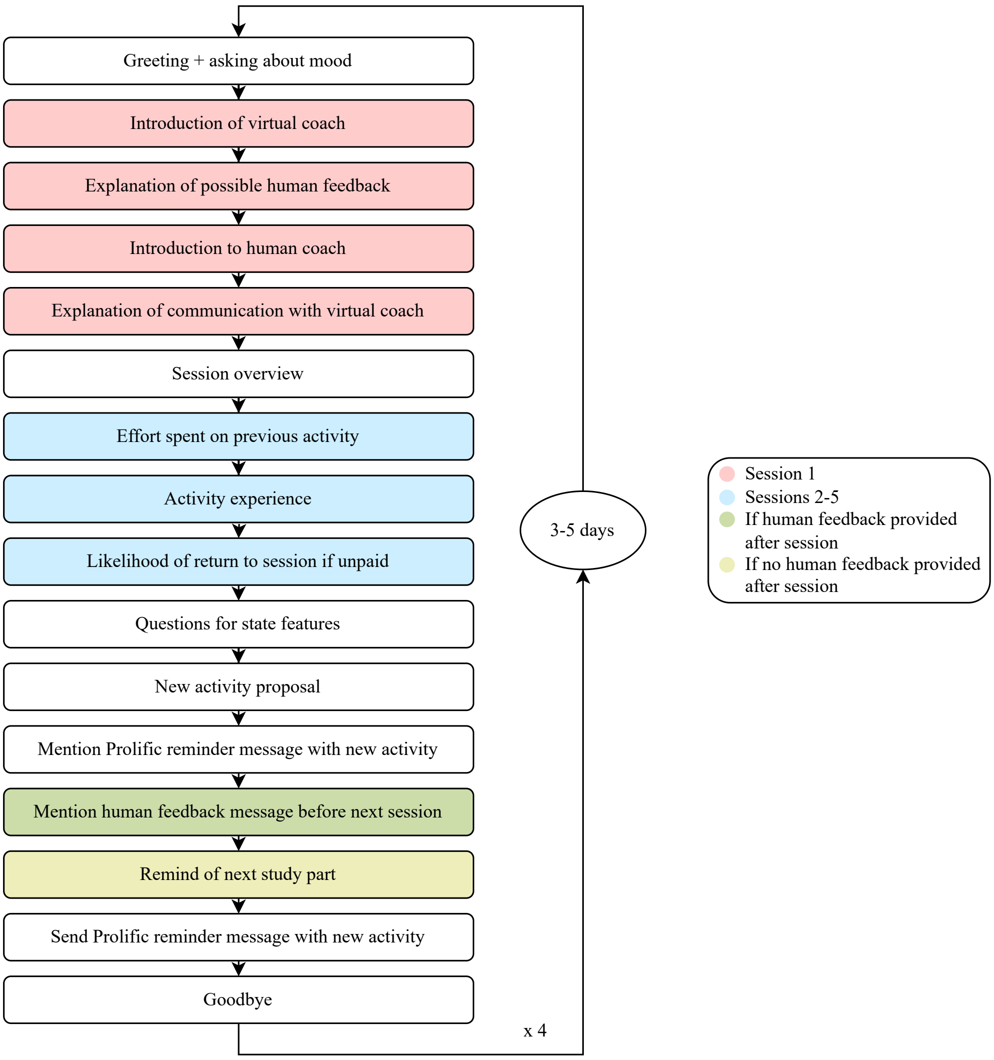

Supplementary Figure 3: Structure of the five conversational sessions with Kai. Participants received reminder messages with the formulations of their new activities on the online crowdsourcing platform Prolific Academic, which is where participants were recruited.

Supplementary Table 3: Preparatory activities for quitting smoking. Titles and formulations from the reminder messages for the 37 preparatory activities for quitting smoking that were used in the study. We also provide the prerequisite activities where applicable as well as refer to sources the activities are based on. For vapers, the formulations were adapted by, for example, replacing "smoking" with "vaping." For an example of how the reminder messages were sent to participants, refer to Supplementary Figure 2.

	Title and formulation	Prerequisite	Sources
1	**Creating motivational slogans/quotes for quitting smoking.** Having strong motivation to quit smoking helps to quit successfully. Before the next session, I thus suggest you take some time to look for a motivational quote or write down something that motivates you to quit smoking. Place this somewhere you can see it every day, such as your fridge or closet door.		Michie et al. [2]

Supplementary Table 3: (continued)

	Title and formulation	Prerequisite	Sources
2	**Creating motivational slogans/quotes for becoming more physically active.** Having strong motivation to become more physically active helps to succeed. Before the next session, I thus suggest you take some time to look for a motivational quote or write down something that motivates you to become more physically active. Place this somewhere you can see it every day, such as your fridge or closet door.		Michie et al. [2]
3	**Testimonial on becoming more physically active.** When preparing for becoming more physically active, it can be useful to learn from other people who have succeeded in becoming more physically active. What goal did they set for themselves? And how did they reach it? Before the next session, I thus recommend you watch this short video in which 5 people describe how they reached their physical activity goals: https://youtu.be/m1MHo9fCTG8. What can you take away from the 5 examples for yourself? Take a few notes on a piece of paper or your phone.		Albers et al. [3], Hizli et al. [4]
4	**Desired future self after quitting smoking - Writing.** Having high aspiration to quit smoking may aid in quitting successfully. Thus, before the next session, I advise you to think about the person that you would like to be once you have successfully quit smoking. For example, you might want to be a “grandfather who can play football with his grandchildren” or a “nurse who can walk up the stairs to the fourth floor without getting out of breath.” Write down everything that comes to your mind.		Albers et al. [5], Meijer et al. [6], Penfornis et al. [7]
5	**Desired future self after becoming more physically active - Writing.** Quitting smoking may be easier if you become more physically active (e.g., take walks, swim, or go running). One important step for this is to have a high ambition to become more physically active. Thus, before the next session, I advise you to think about the person that you would like to be once you have become more physically active. For example, you might want to be a “grandfather who can play football with his grandchildren” or a “nurse who can walk up the stairs to the fourth floor without getting out of breath.” Write down everything that comes to your mind.		Albers et al. [5], Meijer et al. [6], Penfornis et al. [7]

Supplementary Table 3: (continued)

	Title and formulation	Prerequisite	Sources
6	**Reasons for quitting smoking.** Quitting smoking has many benefits. Think, for example, of improved physical fitness, healthier skin, and lower expenses. To help you quit smoking, it can be useful to write down why you want to quit. This can increase your aspiration to quit smoking, which may aid in quitting successfully. So, before the next session, I advise you to identify and write down reasons why you want to stop smoking. After writing them down, think about which reasons are most important to you and order them accordingly.		Albers et al. [5], Michie et al. [8], Trimbos Instituut [9]
7	**Reasons for becoming more physically active.** Quitting smoking may be easier if you become more physically active (e.g., take walks, swim, or go running). One crucial step for this is to have a strong desire to become more physically active. Therefore, before the next session, I advise you to identify and write down reasons why you want to become more physically active. After writing them down, think about which reasons are most important to you and order them accordingly.		Albers et al. [5], Michie et al. [8]
8	**Personal rule for not smoking.** Having strong determination to refrain from smoking may help to quit successfully. So, before the next session, I advise you to take some time to create a personal rule that helps you to refrain from smoking. Possible examples include "Not a puff - no matter what," "Say no to smoking, yes to life" or "Smoking is NOT an option." Write down your rule on a piece of paper and repeat it to yourself 3 times. Put the piece of paper with your rule somewhere you can see it every day.		Albers et al. [5], Michie et al. [8]
9	**Personal rule for becoming more physically active.** Being more physically active (e.g., taking walks, swimming, or going running) may aid you to stop smoking. One important aspect for this is to have strong resolve to become more physically active. So, before the next session, I advise you to take some time to create a personal rule that helps you to become more physically active. Possible examples include "10 squats - no matter what," "Say no to sitting, yes to life" or "Driving to the grocery store is NOT an option." Write down your rule on a piece of paper and repeat it to yourself 3 times. Put the piece of paper with your rule somewhere you can see it every day.		Albers et al. [5], Michie et al. [8]

Supplementary Table 3: (continued)

	Title and formulation	Prerequisite	Sources
10	**How friends and/or family will receive one's desired future self after quitting smoking.** People are social creatures. This means that what we do is noticed by others around us, and they can react to it. To help you quit smoking, it can be useful to imagine how people who are important to you will receive your non-smoker future self. This can boost your confidence. The reactions of others, such as friends, colleagues, and family, to your future self can be positive, but they could also be negative. It is good to be prepared for both possibilities. Before the next session, I suggest you grab a pen and paper and answer these 4 questions: 1) How would the people who are important to you react to the new you, who has quit smoking? 2) How would you feel about their reactions? 3) How would you react if the people who are important to you react positively to the new you? 4) How would you react if the people who are important to you react negatively to the new you?	4 or 24	Mercken et al. [10], Perfect Fit project
11	**How friends and/or family will receive one's desired future self after becoming more physically active.** As social beings, our actions are observed by those around us, who may react in various ways. To boost your confidence when preparing for becoming more physically active, it can be beneficial to envision how those who are important to you will respond to your future physically active self. This can be your colleagues, friends, family, or neighbors, for example. While their reactions may be positive, they could also be negative. So it is wise to anticipate and prepare for both possible outcomes. Before the next session, I suggest you grab a pen and paper and answer these 4 questions: 1) How would the people who are important to you react to the new you, who has become more physically active? 2) How would you feel about their reactions? 3) How would you react if the people who are important to you react positively to the new you? 4) How would you react if the people who are important to you react negatively to the new you?	5 or 25	Mercken et al. [10], Perfect Fit project

Supplementary Table 3: (continued)

	Title and formulation	Prerequisite	Sources
12	**Focusing on past successes for quitting smoking.** To increase your confidence that you will succeed in quitting smoking, it can help to think back to previous successes. Have you ever quit smoking before or reduced the number of times you smoked? Maybe you once only had one instead of two cigarettes after dinner. Every success in quitting smoking counts! Before the next session, I suggest you take some time to think about your previous successes and make a list on a piece of paper. Take a few minutes to make your list before moving on. Then take a closer look at your list. Try to think about what strengths you have that helped you to achieve these things. Write these strengths down on your list so you do not forget them. You can also hang or place your list somewhere in your home so that you are reminded of your successes and strengths more often. The list shows that you can be proud of yourself.		Michie et al. [2], Perfect Fit project, Stichting Stop Bewust [11]
13	**Focusing on past successes for becoming more physically active.** Reflecting on past successes can boost your confidence in successfully becoming more physically active. Before the next session, I suggest you think about when you succeeded in being more physically active. Have you ever taken the bike instead of the car, or taken the stairs instead of the escalator? Every small victory counts! Take a few minutes to make your list before moving on. Then take a closer look at your list. Try to think about what you did that helped you to achieve these things. Write it down on your list so you do not forget it. You can also hang or place your list somewhere in your home so you are reminded of your successes more often. These successes show that you can be proud of yourself.		Michie et al. [2], Perfect Fit project, Stichting Stop Bewust [11]
14	**Role model for others by quitting smoking.** Many people want to quit smoking for other people, such as children or friends. Thinking about how quitting smoking makes you a role model by doing something good for others can motivate you during difficult moments in your quitting journey. I, therefore, recommend you think about how quitting smoking makes you a role model for others before the next session. Grab a pen and a piece of paper and write down your thoughts. Or maybe a picture can help you capture your thoughts.		Perfect Fit project, Trimbos Instituut [9]

Supplementary Table 3: (continued)

	Title and formulation	Prerequisite	Sources
15	**Role model for others by becoming more physically active.** Many people want to become more physically active for other people, such as children or friends. Thinking about how becoming more physically active makes you a role model by doing something good for others can motivate you during difficult moments in your behavior change journey. I, therefore, recommend you think about how becoming more physically active makes you a role model for others before the next session. Grab a pen and a piece of paper and write down your thoughts. Or maybe a picture can help you capture your thoughts.		Perfect Fit project, Trimbos Instituut [9]
16	**Tracking smoking behavior.** Preparing for situations in which you commonly smoke may make it easier to successfully quit smoking. Therefore, I recommend that you record the situations in which you smoke before the next session. Take note of one or two keywords to describe the situation and the number of cigarettes that you smoked. For example, you might note "Lunch break, 2 cigarettes" or "TV, 5 cigarettes." It might be helpful to take these notes on your phone, or you could carry a small piece of paper and pen in your pocket.		Albers et al. [5], Michie et al. [8]
17	**Tracking physical activity behavior.** Becoming more physically active (e.g., taking walks, swimming, or boxing) may make it easier to successfully quit smoking. One important step for becoming more physically active is to know one's current level. This allows one to later set a precise goal and hence to feel more motivated. So, I recommend that you record your current behavior with regard to physical activity before the next session. Try to keep track of how much time you spend 1) sitting, 2) working out and 3) being moderately active (e.g., taking a walk, biking to the grocery store). For this, it might be helpful to keep a piece of paper and pen on your kitchen table, or maybe you have a smartwatch that can record these types of behavior for you.		Albers et al. [5], Michie et al. [8]

Supplementary Table 3: (continued)

	Title and formulation	Prerequisite	Sources
18	**Feared future self when not quitting smoking - Writing.** Having high motivation to quit smoking may aid in quitting successfully. Thus, before the next session, I advise you to think about who you do NOT want to be in the future but might become if you continue to smoke. For example, you might NOT want to be a "mother who dies early of coronary heart disease as her mother did," a "husband who is frowned upon by his wife" or a "man who is dependent on a substance." Write down everything that comes to your mind.		Albers et al. [5], Michie et al. [8]
19	**Feared future self when not becoming more physically active - Writing.** It may be easier to successfully quit smoking if you become more physically active (e.g., swim, take walks, or dance). One crucial step for this is to have high determination to become more physically active. Therefore, before the next session, I advise you to think about who you do NOT want to be in the future but might become if you fail to become more physically active. For example, you might NOT want to be a "mother who dies early of coronary heart disease as her father did," a "daughter who is frowned upon by her mother" or a "man who is dependent on his wife in his everyday life." Write down everything that comes to your mind.		Albers et al. [5], Meijer et al. [6], Penfornis et al. [7]
20	**Feared future self when not quitting smoking - Picture.** Having high motivation to quit smoking may help to quit successfully. So, before the next session, I advise you to think about who you do NOT want to be in the future but might become if you continue to smoke. For example, you might NOT want to be a "mother who dies early of coronary heart disease as her mother did," a "husband who is frowned upon by his wife" or a "man who is dependent on a substance." Then, look for or take a picture that best captures your feared future self. Save or print this picture so that you can see it every day.		Albers et al. [5], Meijer et al. [6], Penfornis et al. [7]

Supplementary Table 3: (continued)

	Title and formulation	Prerequisite	Sources
21	**Feared future self when not becoming more physically active - Picture.** It may be easier to successfully quit smoking if you become more physically active (e.g. exercise, take walks, sit less). One crucial step for this is to have high determination to become more physically active. Therefore, before the next session, I advise you to think about who you do NOT want to be in the future but might become if you fail to become more physically active. For example, you might NOT want to be a "mother who dies early of coronary heart disease as her father did," a "daughter who is frowned upon by her mother" or a "man who is dependent on his wife in his everyday life." Then, look for or take a picture that best captures your feared future self. Save or print this picture so that you can see it every day.		Albers et al. [5], Meijer et al. [6], Penfornis et al. [7]
22	**Visualizing smoking as a battle.** Focusing on your goal of successfully quitting smoking may help you to quit. Thus, before the next session, I advise you to take some time to visualize smoking as a battle. For example, you might see yourself and a cigarette as two boxers in a fighting match. Then imagine yourself winning this battle. Visualize clearly how you win and what it feels like to be the winner. Write down a few words about your winning experience.		Albers et al. [5], Michie et al. [8]
23	**Visualizing becoming more physically active as a battle.** Becoming more physically active (e.g., swimming, taking walks, or dancing) may help you to successfully quit smoking. One important step for this is to focus on the goal of becoming more physically active. Thus, before the next session, I advise you to take some time to visualize becoming more physically active as a battle. For example, you might see yourself and a non-active version of yourself as two boxers in a fighting match. Then imagine yourself winning this battle. Visualize clearly how you win and what it feels like to be the winner. Write down a few words about your winning experience.		Albers et al. [5], Michie et al. [8]

Supplementary Table 3: (continued)

	Title and formulation	Prerequisite	Sources
24	**Desired future self after quitting smoking - Picture.** Having high aspiration to quit smoking may aid in quitting successfully. Thus, before the next session, I advise you to think about the person that you would like to be once you have successfully quit smoking. For example, you might want to be a "strong woman who lives a healthy life" or a "father who is a good role model for his children." Then look for or take a picture that best captures your desired future self. Save or print this picture so that you can see it every day.		Albers et al. [5], Meijer et al. [6], Penfornis et al. [7]
25	**Desired future self after becoming more physically active - Picture.** Quitting smoking may be easier if you become more physically active (e.g., take walks, dance, or swim). One crucial step for this is to have high motivation to become more physically active. Thus, before the next session, I advise you to think about the person that you would like to be once you have become more physically active. For example, you might want to be a "grandfather who can play football with his grandchildren" or a "nurse who can walk up the stairs to the fourth floor without getting out of breath." Then look for or take a picture that best captures your desired future self. Save or print this picture so that you can see it every day.		Albers et al. [5], Meijer et al. [6], Penfornis et al. [7]
26	**Education on sleep.** If you do not smoke, you sometimes sleep worse. This can make it more difficult to remain quit. You can sleep worse, for example, if you have the same caffeine intake (e.g., coffee, tea, energy drinks, chocolate) as before quitting because caffeine is metabolized less quickly once you quit. Before the next session, I thus recommend you watch this short video for a few tips for better sleep: https://www.youtube.com/watch?v=nysjq8VIwI8&ab_channel=EveryMindMatters. How do you plan to use these tips to improve your sleep after quitting smoking? Take a few notes on your phone or a piece of paper.		Perfect Fit project, Trimbos Instituut [9]

Supplementary Table 3: (continued)

	Title and formulation	Prerequisite	Sources
27	**Routines that cause cravings.** Getting fewer cravings to smoke may make it easier to successfully quit smoking. Therefore, before the next session, I advise you to think about routines in your daily life that often cause you to get cravings to smoke. For example, you might have experienced that if you go to bed very late and thus sleep less, you smoke more the next day. Or maybe you have noticed that if you skip your breakfast, you always smoke on your way to work but NOT otherwise. How could you change these routines to reduce or even avoid those cravings? Write down everything that comes to your mind.	16	Albers et al. [5], Michie et al. [8]
28	**Thinking of high-risk situations and how to cope with them.** Preparing for situations in which avoiding smoking is difficult may make it easier to successfully quit smoking. Thus, before the next session, I advise you to think about situations in which you might find it difficult to refrain from smoking. For example, this could be during your lunch break at work, when you meet your best friend, or when you watch TV. How could you deal with these situations so that you do NOT smoke? Write down your plans in a few words.	16	Albers et al. [5], Michie et al. [8]
29	**Alternative behaviors for cravings.** Planning how to resist urges to smoke may make it easier to successfully quit smoking. Therefore, before the next session, I advise you to think about activities that you could do to keep yourself busy when you feel the urge to smoke so that you do NOT smoke. These urges typically last a few minutes; think about something that you could do in the meantime until the urge has passed. For example, you could water your plants, eat a carrot, do 10 push-ups, or do something for another person in need. Write down everything that comes to your mind.		Albers et al. [5], Michie et al. [8]
30	**Progressive muscle relaxation.** Tensing and relaxing areas of the body can reduce cravings and withdrawal symptoms because it is very difficult to feel tense or uptight in a relaxed body. Thus, before the next session, I advise you to watch the following 15-minute video to learn progressive muscle relaxation (which is a way of relaxing your body): https://www.youtube.com/watch?v=ihO02wUzgkc&ab_channel=MarkConnelly. Even if you have already heard of this technique, it might be a good idea to refresh your memory.		Albers et al. [5], Michie et al. [8]

Supplementary Table 3: (continued)

	Title and formulation	Prerequisite	Sources
31	**Breathing exercise.** When you quit smoking, you may feel restless or irritable during the first days or weeks. To help you quit and stay quit, it can help to learn how to manage these nicotine withdrawal symptoms. One way to manage them is through breathing exercises. So, before the next session, I suggest you to watch this 3-minute video to learn how to do box breathing: https://www.youtube.com/watch?v=tEmt1Znux58&ab_channel=SunnybrookHospital. Even if you have already heard of this technique, it might be a good idea to refresh your memory.		National Cancer Institute [12]
32	**Exchanging a passive activity for an active one.** Becoming more physically active (e.g., taking walks, going running, swimming) may help you to successfully quit smoking. One crucial part for this is to think about ways you can incorporate physical activity into your daily life. One way to do this is to exchange a passive activity for an active one. Therefore, before the next session, I advise you to think about ways you could exchange a passive activity for an active one. For example, you could take the stairs instead of the escalator, bike to work instead of taking the bus, or work at a standing desk. Grab a piece of paper or your phone and write down everything that comes to your mind. Which exchange do you want to focus on? Highlight this exchange.		Voedingscentrum [13]
33	**Thinking of solutions to barriers to becoming physically active.** Becoming more physically active (e.g., taking walks, boxing, dancing) may help you to successfully quit smoking. One important step for becoming more physically active is to remove possible obstacles. Thus, before the next session, I advise you to think about things that make it difficult for you to be physically active. For example, this could be that you do NOT have a raincoat to bike to the grocery store when it is raining, that you do NOT want to work out alone, or that you are at work all day and too exhausted by the time that you come home. What are possible solutions to your barriers? For instance, you could buy a raincoat, join a running group, or take a walk during your lunch break at work. Write down everything that comes to your mind.		Albers et al. [5], Alfaifi et al. [14]

Supplementary Table 3: (continued)

	Title and formulation	Prerequisite	Sources
34	**Education on recommended physical activity.** Quitting smoking may be easier if you become more physically active (e.g., swim, take walks, go running). One important step for becoming more physically active is to set a specific goal and thus to feel more aspiration. Therefore, before the next session, I advise you to watch the following 2-minute video on how much and which type of physical activity is recommended: https://www.youtube.com/watch?v=AAPhWbG_zLs&ab_channel=TREKGroup. Then, compare your physical activity behavior to the recommended amounts for the different types of physical activity. Write down which recommended amounts you meet or exceed, and which ones you do NOT meet.	17	Albers et al. [5]
35	**Plan for becoming more physically active.** Becoming more physically active (e.g., taking walks, dancing, swimming) may help you to successfully quit smoking. One crucial part for this is to create a plan for becoming more physically active. Therefore, before the next session, I advise you to think about what you could do to become more physically active. For example, you could get up from your desk after every 30 minutes of sitting, bike to the grocery store, do 10 squats every morning, or join a running group. Write down everything that comes to your mind. Which plan do you want to focus on? Highlight this plan.	17	Albers et al. [5]
36	**Positive diary.** This activity is called "Positive Diary" and helps you think positively and feel good. This can help you quit smoking and become more physically active. In the evening before going to bed, think about the day you had. Write down 2 or 3 things that happened that you are grateful for, happy about, or that went well. For example, "Someone smiled at me in the supermarket," "I did not smoke today," or "I took a nice walk with a friend." Writing down these positive moments can help you feel better, about yourself and about your day. You can write down anything! It does not have to be about being more physically active or quitting smoking, but can be any enjoyable moment from the day, big or small. This will help you to focus on positive things.		Perfect Fit project, Sutton [15]

Supplementary Table 3: (continued)

	Title and formulation	Prerequisite	Sources
37	**Focusing on past success in general.** To increase your confidence that you will succeed in changing your behavior (e.g., quitting smoking), it can help to think back to previous successes. Before the next session, I suggest you take a moment to reflect on times when you succeeded in something or felt satisfied. Have you ever won a competition? Did you cook something delicious recently? Or maybe you learned a new language? Grab a pen and paper and make a list of your success moments. No success is too small to write down! Take a few minutes to make your list before moving on. Then take a closer look at your list. Try to think about what you did that helped you to achieve your successes. Write it down on your list so you do not forget it. You can also hang or place your list somewhere in your home so that you are reminded of your successes more often. The list shows that you can be proud of yourself.		Michie et al. [2], Perfect Fit project, Stichting Stop Bewust [11]

Measures

Supplementary Table 4: Allocation principles in our post-questionnaire and corresponding principles by Persad et al. [1] together with the mean weights assigned to the principles by participants. For each principle, we also quote the allocation preferences initially expressed in free text by a participant who *afterward* assigned a relatively high weight to the principle. This weight afterward assigned to the principle is given in parentheses. Note that since participants expressed their initial allocation preferences in free text *before* they saw and weighted the principles, they might also have assigned a high weight to a principle they had not initially considered. As such, there is not always a clear match between the example quotes and the principles. We provide direct, uncorrected quotes.

	**Allocation principle**	**Principle by Persad et al. [1]**	**Weight**	**Example quote of initial preferences**
Treating people equally				
1	Random	Lottery	9.69%	"Randomly to make it fair I suppose" (100%)
2	Longest time since last human feedback	First-come, first-served	6.45%	"Randomness" (25%)
3	Least amount of human feedback so far	/	6.04%	"Help those first who need the most help but also be equal like give feedback at least once to each person" (20%)
Favoring the worst-off: prioritarianism				
4	Least likely to successfully prepare for quitting [smoking/vaping] without human feedback	Sickest first	13.51%	"When the person seems to be struggling with the program" (70%)
5	Most likely to experience negative consequences of [smoking/vaping] in the future without human feedback	Sickest first	11.82%	"help those who seem struggle more or it is more urgent to quit as soon as possible" (30%)
6	Youngest first	Youngest first	5.31%	"The most urgent cases should take priority over the milder ones ..." (35%)
Maximizing total benefits: utilitarianism				
7	Largest increase in chance of successfully preparing for quitting [smoking/vaping] because of the feedback	Prognosis	16.42%	"The people who are struggling the most should receive the feedback" (100%)

Supplementary Table 4: (continued)

	Allocation principle	Principle by Persad et al. [1]	Weight	Example quote of initial preferences
8	Largest reduction in negative consequences of [smoking/vaping] in the future because of the feedback	Prognosis	14.40%	"I think the virtual coach can use an algorithm to decide who is struggling and in the most need of the human coaches time" (40%)
Promoting and rewarding social usefulness				
9	Largest value to society in the future (e.g., healthcare staff, workers producing influenza vaccine, people who agree to improve their health and thus use fewer resources in the future)	Instrumental value	3.83%	"how much they want to quit smoking and how much effort they put into interacting with kia" (30%)
10	Past usefulness or sacrifice (e.g., past organ donors, people who participated in vaccine research, people who made healthy lifestyle choices that reduced their need for resources in the past)	Reciprocity	3.90%	"virtual coach should prioritize individuals who are making significant progress or facing specific challenges in their journey to quit smoking" (25%)
Respecting autonomy				
11	Highest appreciation of human feedback	/	8.62%	"People who indicated that they wanted human feedback after a session with Kai" (100%)

Participants

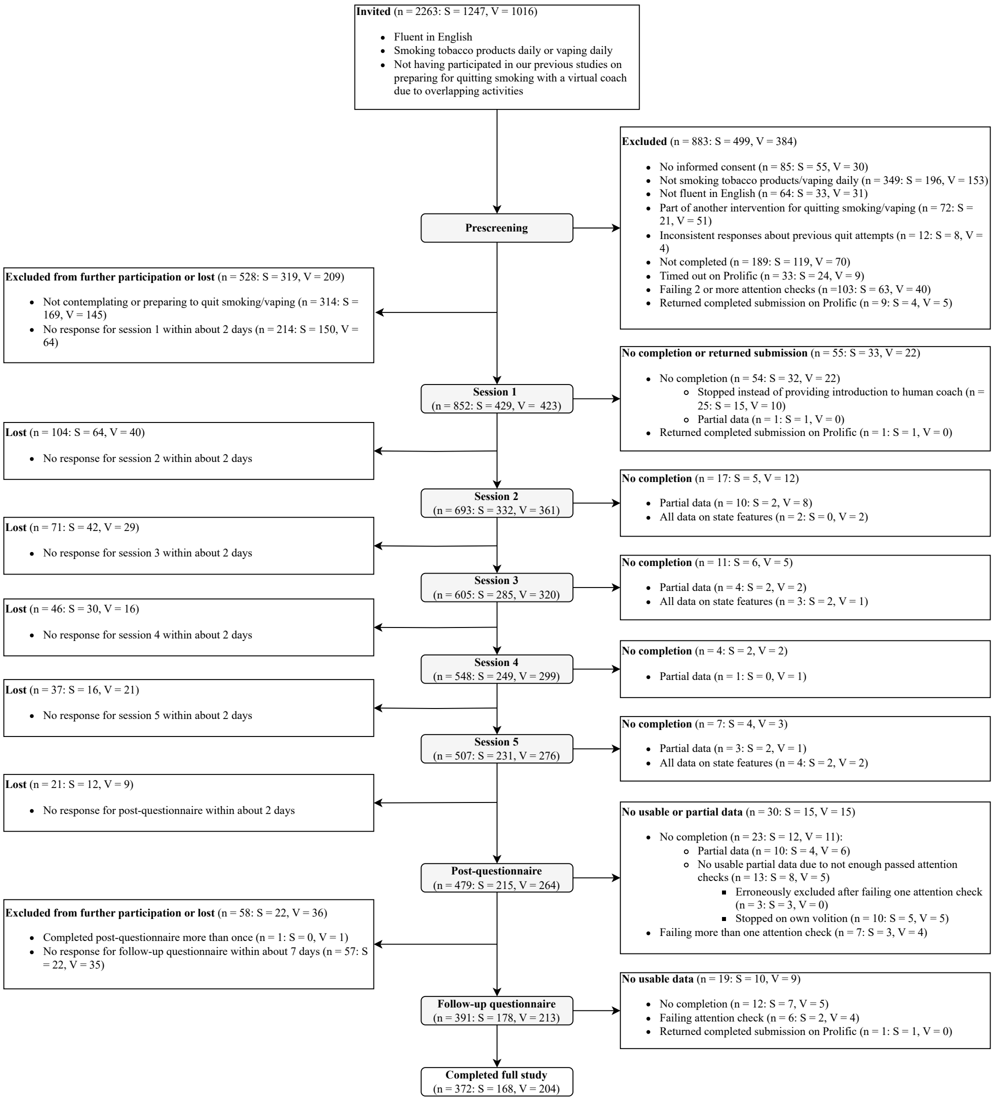

Supplementary Figure 4: Participant flow. Participants on Prolific Academic can return their submissions. Data from the follow-up questionnaire is not included in this paper.

Supplementary Table 5: Characteristics of the 679 participants with at least one interaction sample. People participated in the study as either smokers or vapers.

Characteristic	Value
Age (in years)	
- Mean (SD)	36.30 (11.21)
- Range	19 – 71
Gender	
- Female, n (%)	330 (48.60%)
- Male, n (%)	335 (49.34%)
- Other, n (%)	14 (2.06%)
Highest completed education level	
- No formal qualifications, n (%)	5 (0.74%)
- Secondary education (e.g. GED/GCSE), n (%)	61 (8.98%)
- High school diploma/A-levels, n (%)	139 (20.47%)
- Technical/community college, n (%)	90 (13.25%)
- Undergraduate degree (BA/BSc/other), n (%)	263 (38.73%)
- Graduate degree (MA/MSc/MPhil/other), n (%)	107 (15.76%)
- Doctorate degree (PhD/other), n (%)	9 (1.33%)
- Don't know/not applicable, n (%)	5 (0.74%)
Participation as smoker vs. vaper	
- Smoker, n (%)	352 (51.84%)
- Vaper, n (%)	327 (48.16%)
Smoking/vaping frequency	
- Once a day, n (%)	30 (4.42%)
- 2 – 5 times a day, n (%)	109 (16.05%)
- 6 – 10 times a day, n (%)	136 (20.03%)
- 11 – 19 times a day, n (%)	160 (23.56%)
- More than 20 times a day, n (%)	244 (35.94%)
TTM-stage for becoming physically active	
- Precontemplation, n (%)	24 (3.53%)
- Contemplation, n (%)	182 (26.80%)
- Preparation, n (%)	146 (21.50%)
- Action, n (%)	98 (14.43%)
- Maintenance, n (%)	228 (33.58%)
Weekly exercise amount	
- Never (0 – 60 minutes per week), n (%)	190 (27.98%)
- Sometimes (60 – 150 minutes per week), n (%)	301 (44.33%)
- Often (more than 150 minutes per week), n (%)	187 (27.54%)

Abbreviations: SD, Standard deviation; GED, General educational development; GCSE, General certificate of secondary education; BA, Bachelor of Arts; BSc, Bachelor of Science; MA, Master of Arts; MSc, Master of Science; MPhil, Master of Philosophy; PhD, Doctor of Philosophy; TTM, Transtheoretical model.

Data analysis for RQ2: Long-term effects of optimally allocated human feedback on engagement

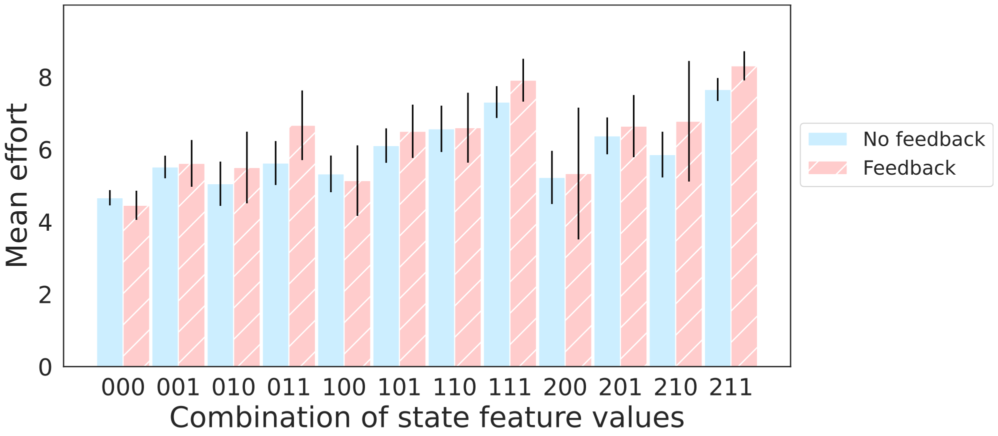

Supplementary Figure 5: Mean effort with 95% credible intervals per action and combination of values for the three selected state features. We denote the values of the three features using this order: 1) perceived importance, 2) self-efficacy, and 3) human feedback appreciation.

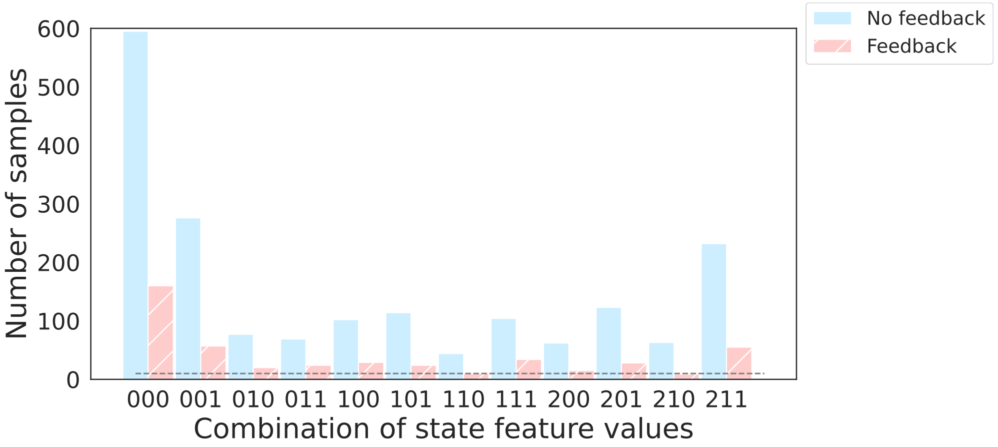

Supplementary Figure 6: Number of samples per action and combination of values for the three selected state features. We denote the values of the three features using this order: 1) perceived importance, 2) self-efficacy, and 3) human feedback appreciation. When we have less than 10 samples (dashed horizontal line) for a feature value combination and action, we impute with the mean effort spent on preparatory activities for the effort prediction in our RL-based analyses.

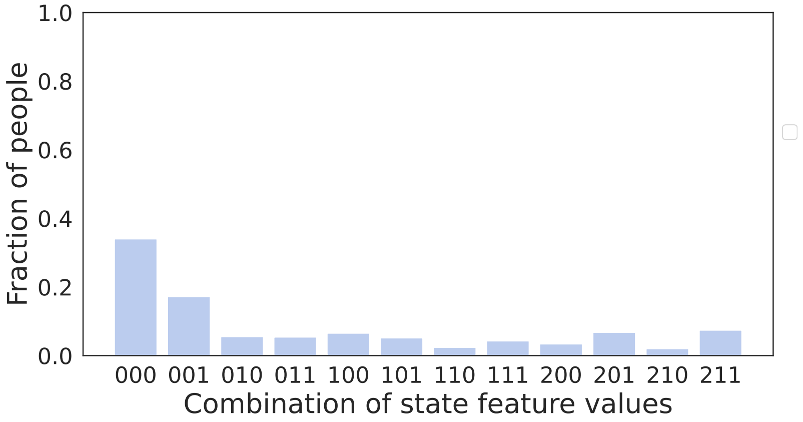

Supplementary Figure 7: Initial distribution of people across the 12 base states observed in the first session of our longitudinal study. We denote the values of the three features using this order: 1) perceived importance, 2) self-efficacy, and 3) human feedback appreciation.

Data analysis for RQ3: Effect of different ethical allocation principles on human feedback received by smoker subgroups

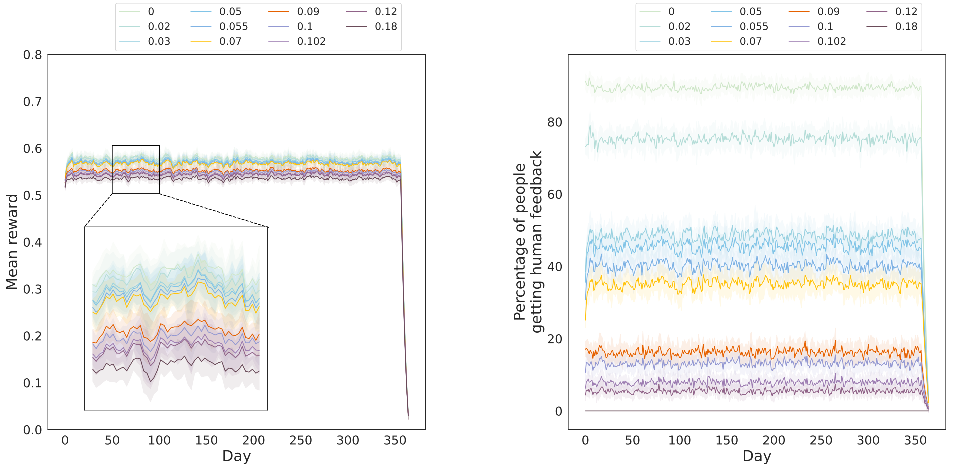

(a) (b)

Supplementary Figure 8: Impact of different human feedback costs on the reward and amount of allocated human feedback in our potential live application. (a) Mean reward and (b) percentage of people receiving feedback per time step for different human feedback costs in our potential live application.

Summary for lay audience

Motivated by previous work indicating that including human support can make people feel more accountable and satisfied with an eHealth intervention, we examined in this work how effective it is to add support from a human coach to a virtual coach-based intervention in which people do preparatory activities (e.g., envision desired future self, track smoking behavior, learn a breathing exercise) to prepare for successful smoking cessation. Adding extensive human support would undermine one of the goals of eHealth interventions, namely, the reliance on scarce and expensive healthcare professionals. We thus focused on relatively low-cost human support, in our case feedback messages written by Master's students in Psychology. Using data from a crowdsourcing study in which 679 daily smokers and vapers interacted with a text-based virtual coach in up to five sessions spread over about two weeks, we analyzed psychological, economic, and ethical factors that play a role when allocating this support. We find that having received human feedback increases both the retention and the engagement with activities assigned by the virtual coach. However, there are also situations where not giving feedback is better in the long run. When providing human feedback is expensive, it is most effective in the long run to give this feedback to people who perceive preparing to quit as not that important and at the same time have high self-efficacy for preparing to quit. Notably, while it may seem intuitive to allocate limited human feedback to those who would benefit most from it, it is, in fact, only one of several ethical principles for allocating scarce medical resources. Alternatives include allocating feedback to those who want it most or to individuals who have the lowest likelihood of successfully quitting without such support. Our analysis of a "standard" model for allocating human feedback that optimizes the overall population-level benefit over time shows that such a model prioritizes people who want feedback and are already doing well. While we show that we can include other allocation principles in the model to favor other smoker subgroups, we find that these principles are often interdependent. For example, since we observed that people who are not doing well tend to not want human feedback and benefit less from it, giving more feedback to them means doing worse in respecting people's autonomy and maximizing the overall benefit. As different smoker subgroups benefit depending on the chosen allocation principles, our findings show that moral decisions are unavoidable when human support is limited.

Supplementary References

[1] Persad, G., Wertheimer, A., Emanuel, E.J.: Principles for allocation of scarce medical interventions. The Lancet **373**(9661), 423–431 (2009) https://doi.org/10.1016/S0140-6736(09)60137-9

[2] Michie, S., Ashford, S., Sniehotta, F.F., Dombrowski, S.U., Bishop, A., French, D.P.: A refined taxonomy of behaviour change techniques to help people change their physical activity and healthy eating behaviours: the calo-re taxonomy. Psychology & Health **26**(11), 1479–1498 (2011) https://doi.org/10.1080/08870446.2010.540664

[3] Albers, N., Hizli, B., Scheltinga, B.L., Meijer, E., Brinkman, W.-P.: Setting physical activity goals with a virtual coach: Vicarious experiences, personalization and acceptance. Journal of Medical Systems **47**, 15 (2023) https://doi.org/10.1007/s10916-022-01899-9

[4] Hizli, B., Albers, N., Brinkman, W.-P.: Data and code underlying the master thesis: Goal-setting dialogue for physical activity with a virtual coach. 4TU.ResearchData (2022). https://doi.org/10.4121/20047328

[5] Albers, N., Neerincx, M.A., Penfornis, K.M., Brinkman, W.-P.: Users' needs for a digital smoking cessation application and how to address them: A mixed-methods study. PeerJ **10**, 13824 (2022) https://doi.org/10.7717/peerj.13824

[6] Meijer, E., Gebhardt, W.A., Laar, C., Putte, B., Evers, A.W.: Strengthening quitter self-identity: An experimental study. Psychology & Health **33**(10), 1229–1250 (2018) https://doi.org/10.1080/08870446.2018.1478976

[7] Penfornis, K.M., Gebhardt, W.A., Rippe, R.C., Van Laar, C., Putte, B., Meijer, E.: My future-self has (not) quit smoking: An experimental study into the effect of a future-self intervention on smoking-related self-identity constructs. Social Science & Medicine **320**, 115667 (2023) https://doi.org/10.1016/j.socscimed.2023.115667

[8] Michie, S., Brown, J., Geraghty, A.W., Miller, S., Yardley, L., Gardner, B., Shahab, L., McEwen, A., Stapleton, J.A., West, R.: Development of stopadvisor: a theory-based interactive internet-based smoking cessation intervention. Translational Behavioral Medicine **2**(3), 263–275 (2012) https://doi.org/10.1007/s13142-012-0135-6

[9] Trimbos Instituut: De Stopcoach (2023). https://www.trimbos.nl/aanbod/interventies/de-stopcoach/

[10] Mercken, L., Candel, M., Van Osch, L., Vries, H.: No smoke without fire: The impact of future friends on adolescent smoking behaviour. British Journal of Health Psychology **16**(1), 170–188 (2011) https://doi.org/10.1348/

135910710X531608

[11] Stichting Stop Bewust Accessed in 2023. https://stichtingstopbewust.nl/

[12] National Cancer Institute: Handling Nicotine Withdrawal and Triggers When You Decide To Quit Tobacco. Accessed in 2023 (2022). https://www.cancer.gov/about-cancer/causes-prevention/risk/tobacco/withdrawal-fact-sheet

[13] Voedingscentrum: Zo maak je een eigen Eetwissel. Accessed in 2023. https://www.voedingscentrum.nl/nl/thema/eetwissel/zo-maak-je-een-eigen-eetwissel.aspx

[14] Alfaifi, Y., Grasso, F., Tamma, V.: An ontology of psychological barriers to support behaviour change. In: Kostkova, P., Grasso, F., Castillo, C., Mejova, Y., Bosman, A., Edelstein, M. (eds.) Proceedings of the 2018 International Conference on Digital Health, DH 2018, Lyon, France, April 23-26, 2018, pp. 11–15. ACM, ??? (2018). https://doi.org/10.1145/3194658.3194680

[15] Sutton, J.: What Is a Thought Diary in CBT? 5 Templates and Examples. Accessed in 2023 (2021). https://positivepsychology.com/thought-diary/
